# Supplementary material for: Evolution of chromosome-arm aberrations in breast cancer through genetic network rewiring
Source: Cell Rep. 2024 Mar 22;43(4):113988. doi: 10.1016/j.celrep.2024.113988 (PMC11063629; doi:10.1016/j.celrep.2024.113988)
Supplement: Document S1. Figures S1–S5 [file mmc1.pdf]

Cell Reports, Volume *43*

Supplemental information

Evolution of chromosome-arm aberrations

in breast cancer through genetic network rewiring

Elena Kuzmin, Toby M. Baker, Tom Lesluyes, Jean Monlong, Kento T. Abe, Paula P. Coelho, Michael Schwartz, Joseph Del Corpo, Dongmei Zou, Genevieve Morin, Alain Pacis, Yang Yang, Constanza Martinez, Jarrett Barber, Hellen Kuasne, Rui Li, Mathieu Bourgey, Anne-Marie Fortier, Peter G. Davison, Atilla Omeroglu, Marie-Christine Guiot, Quaid Morris, Claudia L. Kleinman, Sidong Huang, Anne-Claude Gingras, Jiannis Ragoussis, Guillaume Bourque, Peter Van Loo, and Morag Park

Figure S1

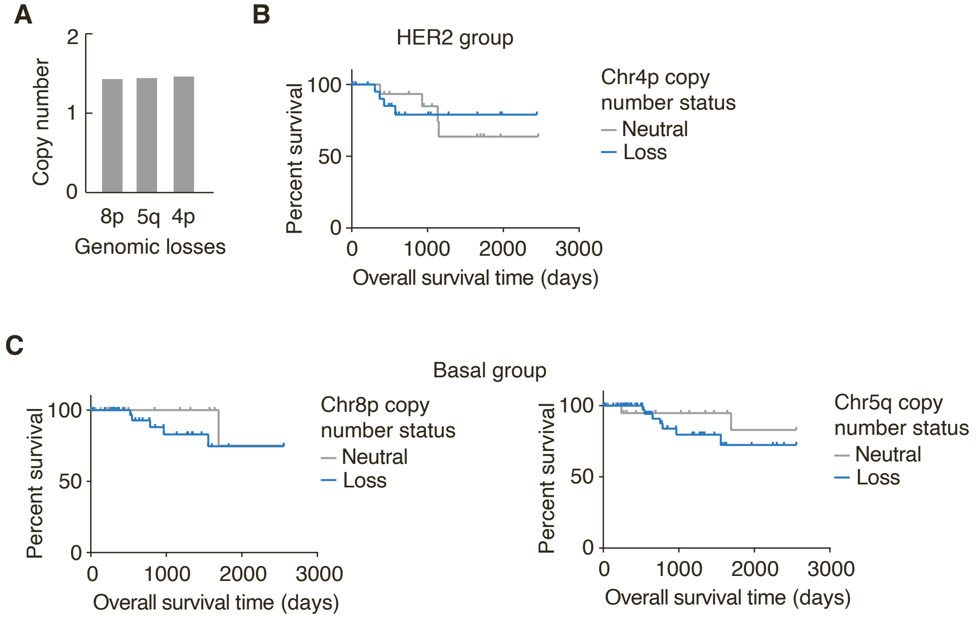

Figure S1. Recurrent large chromosomal deletions in breast cancer. (A) Copy number was obtained from the TCGA segmented mean showing that frequently recurrent large chromosomal deletions in basal breast cancer are hemizygous, n = 91. **(B)** Overall survival of Her2 breast cancer patients with copy neutral and deletion status of chr4p shows no difference between groups, n = 55. **(C)** Overall survival of basal breast cancer patients with copy neutral and deletion status of chr8p and chr5q shows a trends towards a worse survival of patients with these chromosome arm losses (chr 8p loss $p = 0.5$, chr5q loss $p = 0.4$ as assessed by long rank test; n = 91).

Figure S2

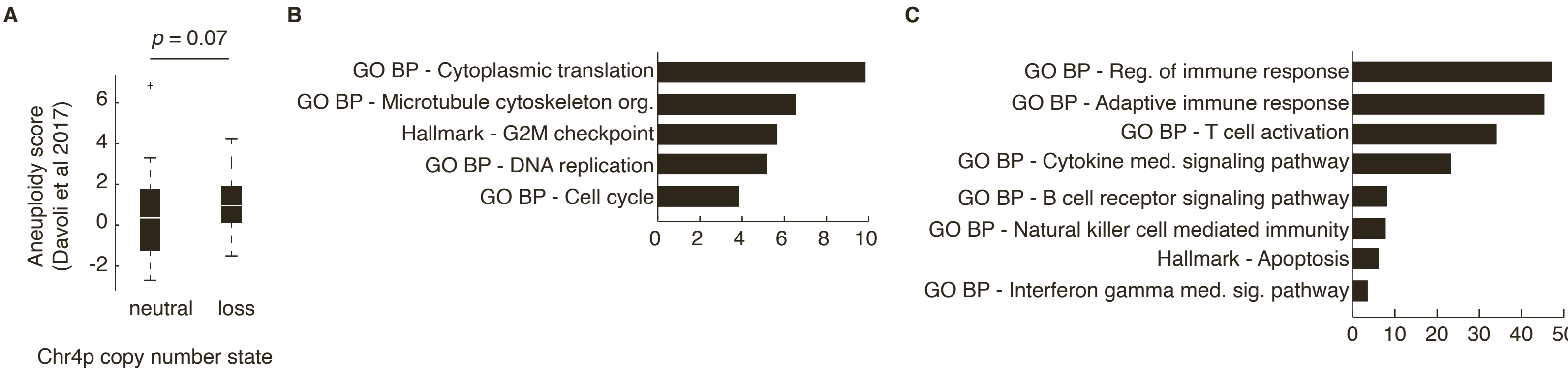

Figure S2. Aneuploidy in basal breast cancer with different chr4p copy number states. (A) Aneuploidy score as quantified by Chrom.Arm.SCNA.Level median reported by (Davoli et al)20 shows no statistically significant difference in aneuploidy between chr4p copy neutral vs deletion basal breast cancer samples. Significance was assessed using Wilcoxon rank sum test Gene Set Enrichment Analysis (GSEA) showing representative terms that are enriched for genes displaying **(B)** elevated or **(C)** decreased expression due to chr4p loss in TCGA basal breast cancer after correcting for aneuploidy as scored in **(A)**.

Figure S3

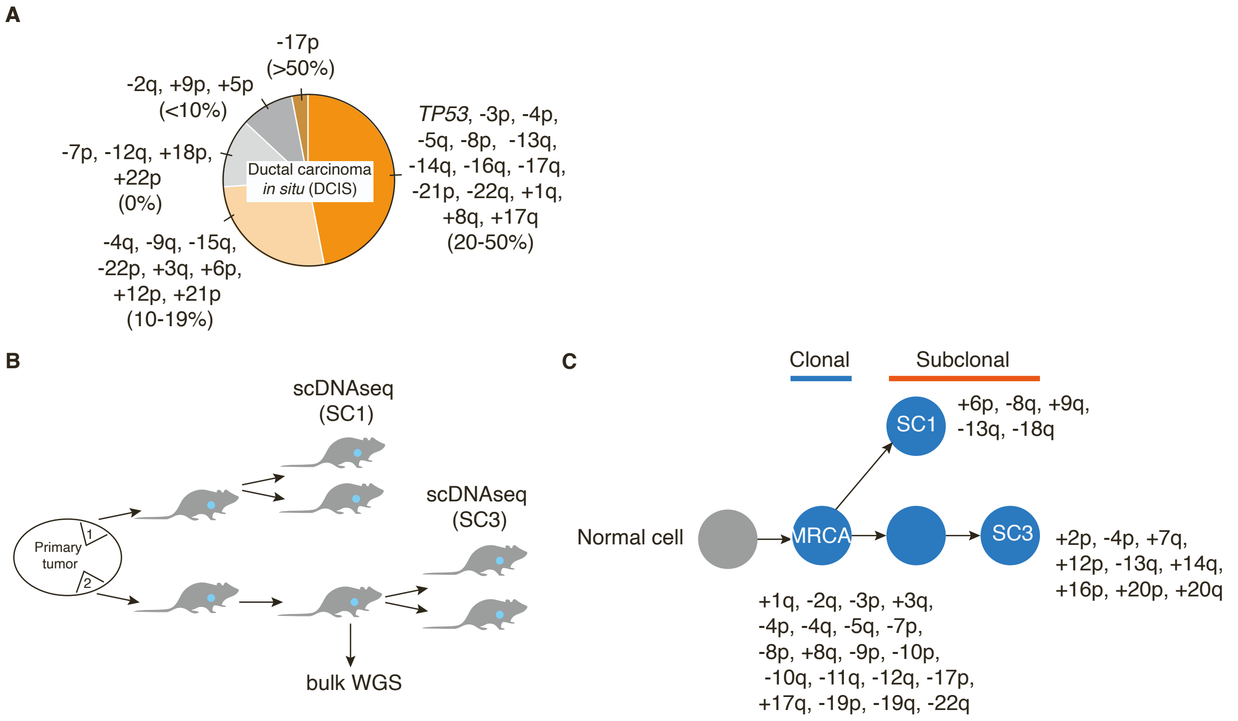

Figure S3. Frequency of genomic events in other datasets. (A) Genetic events from clonal and variable/constant regions of the timing analysis presented in Figure 2B were detected in Ductal Carcinoma *in situ* (DCIS) from a previous study (Lips et al)31. Frequency of patient samples from a total of 95 is shown in brackets. **(B)** Schematic of PDX generation, which was used for bulk WGS and scDNAseq. Single cell DNA sequencing was conducted on four GCRC1735 PDX samples. Two different locations within the primary tumor were biopsied, cryopreserved and propageted in NOD-SCID mice. Two mice were engrafted using a fragment derived from one location from passage 2 in the PDX and two mice were engrafted using a fragment drived from another location from passage 3 in the PDX. **(C)** CHISEL (Zaccaria et al)91 was used to generate an evolutionary timeline showing that chr4p loss is an early event in basal breast cancer PDX progression.

Figure S4

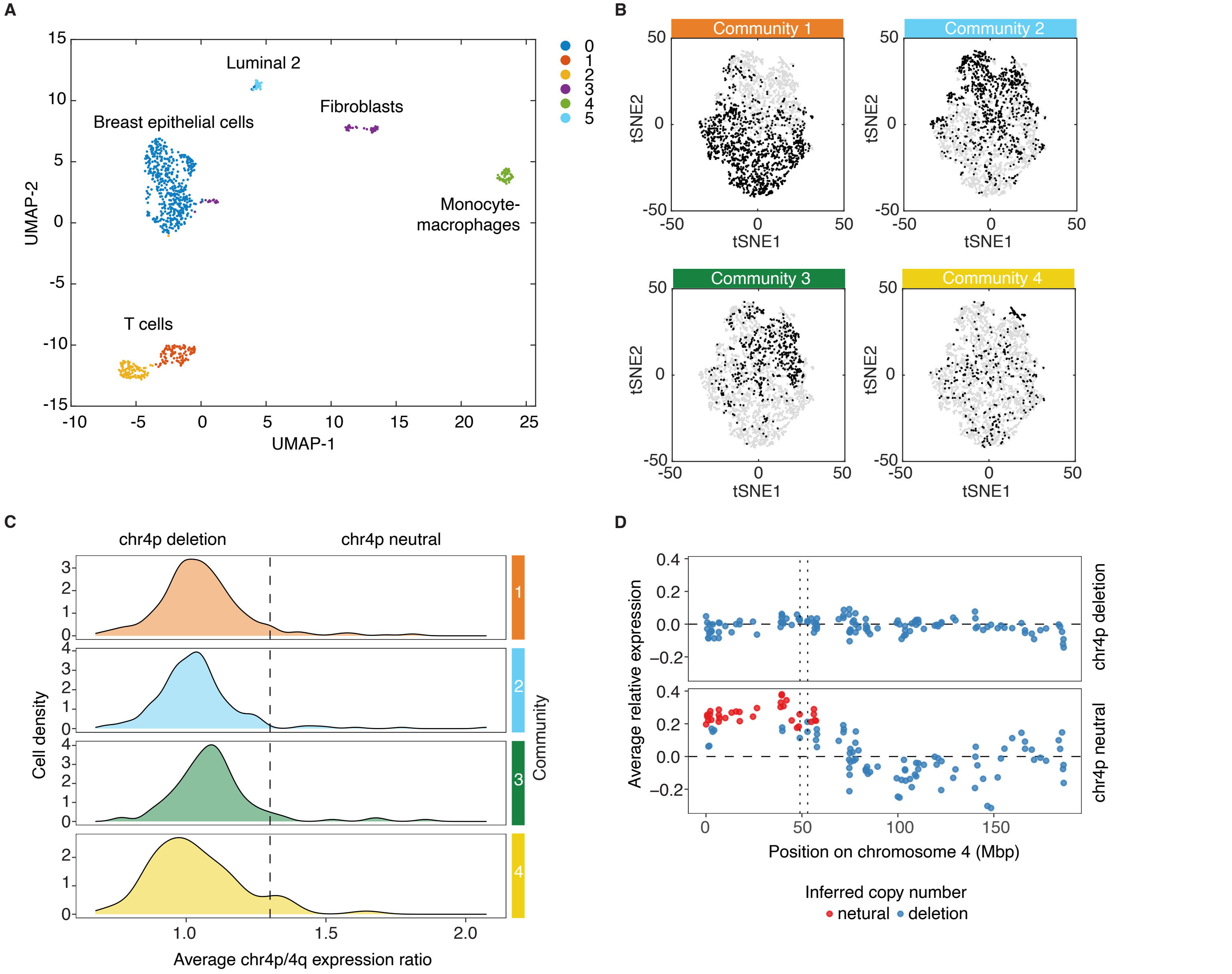

Figure S4. Inferring copy number aberrations from scRNAseq. (A) scRNAseq of normal breast epithelial cells. UMAP plot of single cell RNA seq data from two reduction mammoplasty samples. Clusters were annotated using previously defined cell type markers. **(B)** Overlapping inferred copy number communities with scRNAseq gene expression clusters. scRNAseq map as reported in a previous study is used to annotate single cells with inferred copy number communities as analyzed in this study. Single cells coloured in black belong to the specifiied community. **(C)** To infer chr4p-copy neutral state, we computed the average expression between regions in chr4p and 4q (x-axis). A ratio higher than 1 suggests more copies of chr4p, indicating chr4p copy neutral cells since cells in this tumor harbor chr4q loss. The cells are grouped by the CNA communities depicted in Fig. 4A. The dotted line shows the thresholds used to separate chr4p neutral cells from chr4p loss cells. **(D)** Inferred copy number aberrations shows a clear and consistent signal for chr4p in cells as computed in panel C. The x-axis shows the position across chromosome 4, with the centromere marked with vertical dotted lines. The y-axis shows the average expression of the region relative to the expression along chr4p across all cells attributed to genomic copy number state (higher: neutral, lower: deletion).

Figure S5

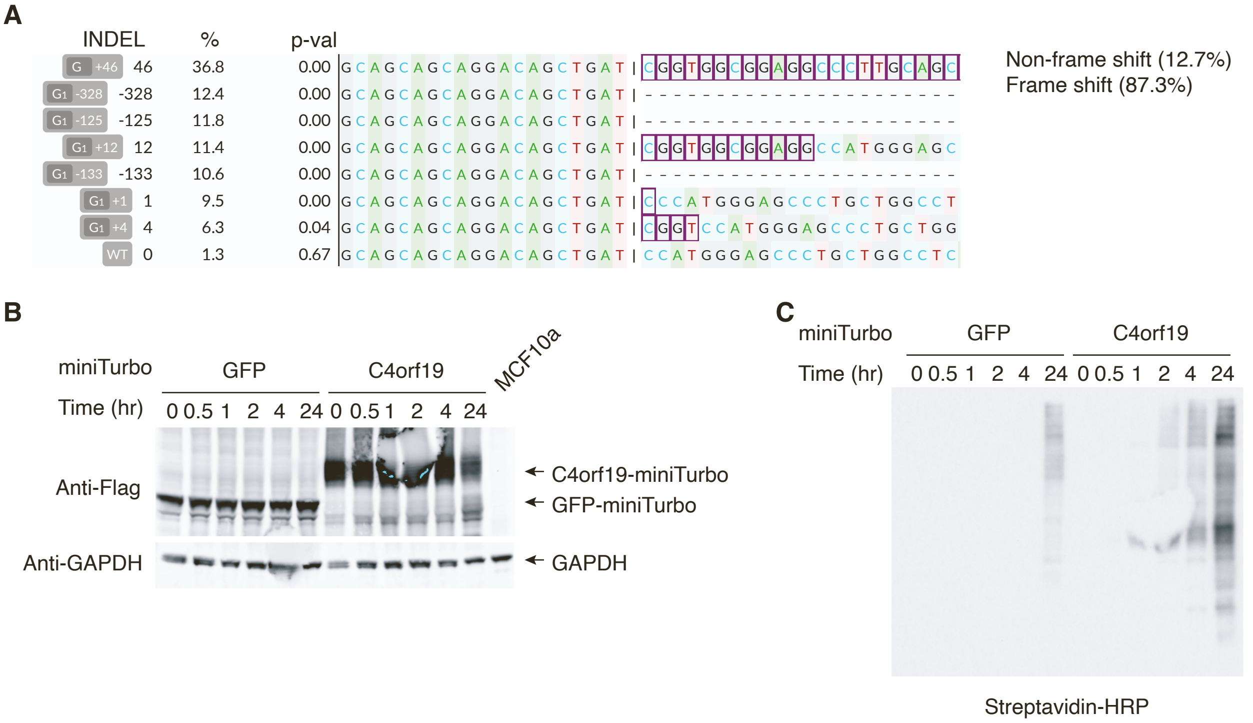

Figure S5. *C4orf19* functional characterization. (A) *C4orf19* CRISPR-Cas9 gene editing efficiency as reported by DECODR. INDEL refers to indel distribution with percentage of reads with a specific indel noted in (%) with the corresponding p-value. **(B)** miniTurboID C4orf19 protein bait expression in MCF10a. MCF10a cells stably expressing miniTurboID bait proteins: C4orf19-3XFLAG-miniTurbo and GFP-3XFLAG-miniTurbo control were induced with 0.5 μg/ml doxycycline for designated time. Protein bait expression was assessed using Anti-Flag antibody. GAPDH protein level served as the loading control, n = 3. **(C)** Biotinylation level. Biotin labeling was induced with 0.5 μg/ml doxycycline and 40 μM biotin for designated time. Optimal biotinylation was achieved 4 hr post induction, n = 3.
