# Supplementary material for: Evolution of chromosome-arm aberrations in breast cancer through genetic network rewiring
Source: Cell Rep. 2024 Mar 22;43(4):113988. doi: 10.1016/j.celrep.2024.113988 (PMC11063629; doi:10.1016/j.celrep.2024.113988)
Supplement: Document S2. Article plus supplemental information [file mmc7.pdf]

Cell Reports

Article

Evolution of chromosome-arm aberrations in breast cancer through genetic network rewiring

Graphical abstract

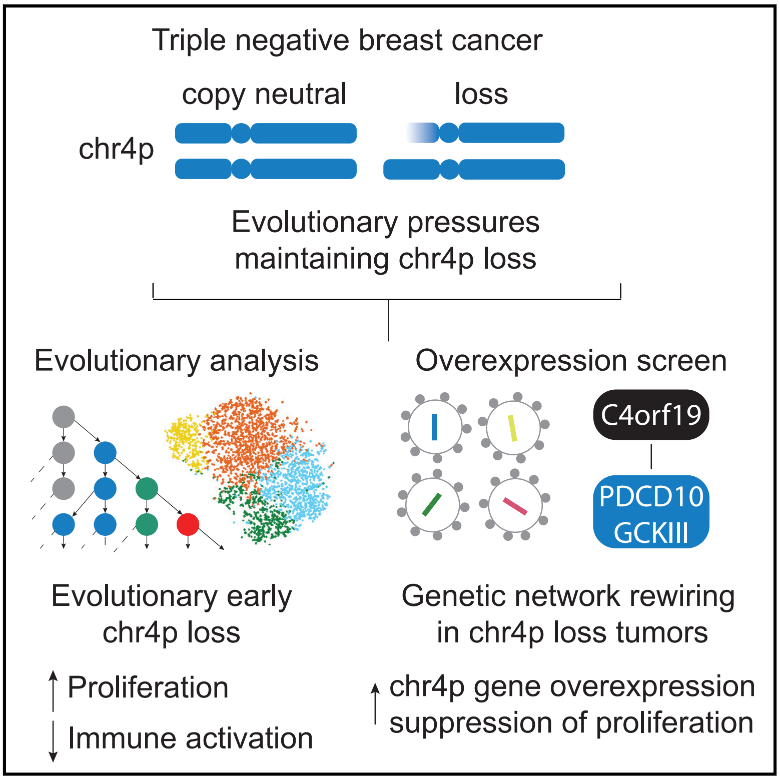

Authors

Elena Kuzmin, Toby M. Baker, Tom Lesluyes, ..., Guillaume Bourque, Peter Van Loo, Morag Park

Correspondence

elena.kuzmin@mcgill.ca (E.K.), morag.park@mcgill.ca (M.P.)

In brief

Kuzmin et al. report that chromosome 4p loss evolves early in triple-negative breast cancer (TNBC) and is associated with enhanced proliferation. *C4orf19* (*PGCKA1*) is a tumor suppressor. Certain regions, including chr4p, suppress proliferation when overexpressed, differentially implicating network rewiring. This study illuminates the early emergence of complex aneuploid karyotypes in TNBC.

Highlights

- Chr4p loss evolves early in TNBC
- Chr4p loss enhances growth
- *C4orf19* (*PGCKA1*) tumor suppressor

Kuzmin et al., 2024, Cell Reports *43*, 113988
April 23, 2024 © 2024 The Authors. Published by Elsevier Inc.
https://doi.org/10.1016/j.celrep.2024.113988

CellPress

Cell Reports

CellPress OPEN ACCESS

Article

Evolution of chromosome-arm aberrations in breast cancer through genetic network rewiring

Elena Kuzmin,[1,2,21,22,23,*] Toby M. Baker,[4] Tom Lesluyes,[4] Jean Monlong,[5,6] Kento T. Abe,[7,8] Paula P. Coelho,[1,2] Michael Schwartz,[1,2] Joseph Del Corpo,[3] Dongmei Zou,[1] Genevieve Morin,[1,2] Alain Pacis,[6,9] Yang Yang,[5] Constanza Martinez,[1,10,11] Jarrett Barber,[7,12,13,14] Hellen Kuasne,[1] Rui Li,[5,6] Mathieu Bourgey,[6,9] Anne-Marie Fortier,[1] Peter G. Davison,[15,16] Atilla Omeroglu,[10] Marie-Christine Guiot,[10] Quaid Morris,[7,12,13,14,17] Claudia L. Kleinman,[5,18] Sidong Huang,[1,2,5] Anne-Claude Gingras,[7,8] Jiannis Ragoussis,[5,6] Guillaume Bourque,[5,6,9] Peter Van Loo,[4,19,20] and Morag Park[1,2,11,*]

[1]Rosalind and Morris Goodman Cancer Institute, Montreal, QC H3A 1A3, Canada
[2]Department of Biochemistry, McGill University, Montreal, QC H3G 1Y6, Canada
[3]Department of Biology, Concordia University, Montreal, QC H4B 1R6, Canada
[4]The Francis Crick Institute, NW1 1AT London, UK
[5]Department of Human Genetics, McGill University, Montreal, QC H3A 0C7, Canada
[6]McGill Genome Centre, Montreal, QC H3A 0G1, Canada
[7]Department of Molecular Genetics, University of Toronto, Toronto, ON M5S 1A8, Canada
[8]Lunenfeld-Tanenbaum Research Institute, Mount Sinai Hospital, Sinai Health, Toronto, ON M5G 1X5, Canada
[9]Canadian Centre for Computational Genomics (C3G), McGill University, Montreal, QC H3A 0G1, Canada
[10]Department of Pathology, McGill University, Montreal, QC H3A 2B4, Canada
[11]Gerald Bronfman Department of Oncology, McGill University, Montreal, QC H4A 3T2, Canada
[12]Vector Institute, Toronto, ON M5G 1M1, Canada
[13]Ontario Institute for Cancer Research, Toronto, ON M5G 0A3, Canada
[14]Computational and Systems Biology, Sloan Kettering Institute, New York City, NY 10065, USA
[15]Department of Surgery, McGill University, Montreal, QC H3G 1A4, Canada
[16]McGill University Health Centre, Montreal, QC H4A 3J1, Canada
[17]Gerstner Sloan Kettering Graduate School of Biomedical Sciences, Memorial Sloan Kettering Cancer Center, New York, NY 10065, USA
[18]Lady Davis Institute for Medical Research, Montreal, QC H3T 1E2, Canada
[19]Department of Genetics, The University of Texas MD Anderson Cancer Center, Houston, TX 77030, USA
[20]Department of Genomic Medicine, The University of Texas MD Anderson Cancer Center, Houston, TX 77030, USA
[21]Present address: Department of Biology, Center for Applied Synthetic Biology, Center for Structural and Functional Genomics, Concordia University, Montreal, QC H4B 1R6, Canada
[22]Present address: Department of Human Genetics, McGill University, Montreal, QC H3A 0C7, Canada
[23]Lead contact
*Correspondence: elena.kuzmin@mcgill.ca (E.K.), morag.park@mcgill.ca (M.P.)
https://doi.org/10.1016/j.celrep.2024.113988

SUMMARY

The basal breast cancer subtype is enriched for triple-negative breast cancer (TNBC) and displays consistent large chromosomal deletions. Here, we characterize evolution and maintenance of chromosome 4p (chr4p) loss in basal breast cancer. Analysis of The Cancer Genome Atlas data shows recurrent deletion of chr4p in basal breast cancer. Phylogenetic analysis of a panel of 23 primary tumor/patient-derived xenograft basal breast cancers reveals early evolution of chr4p deletion. Mechanistically we show that chr4p loss is associated with enhanced proliferation. Gene function studies identify an unknown gene, *C4orf19*, within chr4p, which suppresses proliferation when overexpressed—a member of the PDCD10-GCKIII kinase module we name *PGCKA1*. Genome-wide pooled overexpression screens using a barcoded library of human open reading frames identify chromosomal regions, including chr4p, that suppress proliferation when overexpressed in a context-dependent manner, implicating network interactions. Together, these results shed light on the early emergence of complex aneuploid karyotypes involving chr4p and adaptive landscapes shaping breast cancer genomes.

INTRODUCTION

Breast cancer is a heterogeneous disease comprising several clinical and molecular subtypes. Therapeutic strategies have been devised for patients based on biomarkers, such as hormone (estrogen and progesterone) receptor expression or human epidermal growth factor 2 (HER2) receptor amplification.[1] However, triple-negative breast cancer (TNBC), constituting

© 2024 The Authors. Published by Elsevier Inc.
This is an open access article under the CC BY license (http://creativecommons.org/licenses/by/4.0/).

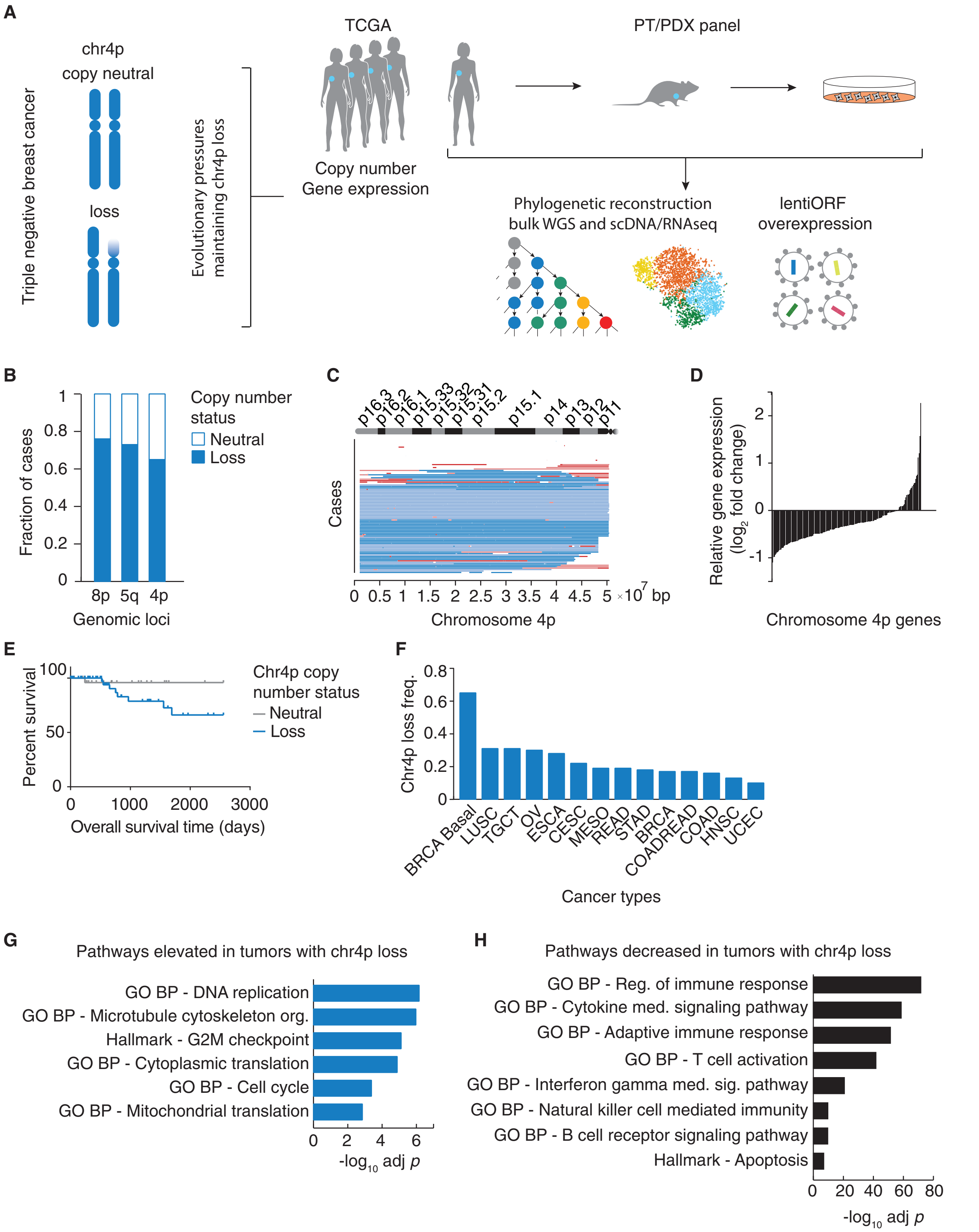

(legend on next page)

10%–20% of all breast cancers, lacks these receptors and thus lacks precision therapies targeting them, and is predominantly treated by chemotherapy. Currently, due to limited therapeutic options, TNBC has the most aggressive behavior and worst prognosis (5-year relative survival percentage), leading to a large percentage of breast cancer deaths.[2–4] The basal breast cancer molecular subtype constitutes ~80% of TNBC and shows a complex mutational spectrum without common oncogenic drivers.[5–7] Notably, basal breast cancers frequently display consistent large chromosomal deletions[8,9] that are thought to play an important role in pathogenesis, the consequences of which are poorly understood.

An important hallmark of cancer cells is genomic instability, which generates mutations and chromosome alterations that confer selective advantage on subclones of cells and lead to their growth and dominance in a local tissue.[10,11] Considerable effort has been invested in identifying which oncogenes or tumor-suppressor genes, through the gain or loss of function, respectively, drive cancer development.[10] With the advent of genomic technologies, it has become possible to generate a detailed map of genetic changes in cancer.[6,12–14] Genomic analyses revealed that chromosome-arm somatic copy-number aberrations are more common than whole-chromosome somatic copy-number aberrations, and certain chromosomal arms are preferentially lost or gained, suggesting that these events are selected because they are advantageous during cancer progression.[15,16] Evolutionary analyses of Pan-Cancer Analysis of Whole Genomes (PCAWG) data on 38 types of cancer showed that chromosomal-arm copy-number losses occur early and typically precede gains, indicating their selective advantage in tumor onset and progression.[17] Recent findings suggest that chromosomal-arm aberrations occur in bursts, enabling genome diversification and preferential clonal expansion in TNBC.[18] Although they have been implicated in some cancers in increasing cell growth[19] and evading immune system detection,[20] the functional consequences of chromosomal-arm deletions remain poorly understood.

We previously established that a large deletion on chromosome 5q in basal breast cancer leads to a loss of function of *KIBRA*, encoding a multi-domain scaffold protein, activating oncogenic transcription factors *YAP/TAZ*.[21] Chromosome 8p loss in breast cancer alters fatty acid and ceramide metabolism, leading to invasiveness and tumor growth under stress conditions due to increased autophagy, thus contributing to resistance to chemotherapeutic agents.[22] Another study found that cooperative effects, resulting from genes co-deleted within a region harboring *TP53* on chromosome 17p, lead to more aggressive lymphoma than individual mutations.[23] Interestingly, a recent study reported differences in specific chromosome-arm losses, such as chr3p loss, which positively correlated with immune signatures, suggesting that specific chromosomal regions can exert selective pressures rather than overall aneuploidy level.[24]

In this study, we identified chr4p loss as a frequently recurrent chromosome-arm loss in the basal subtype of breast cancer and established that this occurred as an early clonal event functionally associated with an enhanced proliferative state (Figure 1A). Scanning genes on chr4p by functional assays, we identified genes whose elevated expression suppressed proliferation in human breast epithelial cells. This included an unknown gene, *C4orf19*, within chr4p, which we show suppresses proliferation and is a member of the programmed cell death 10 (PDCD10)-germinal center kinase III (GCKIII) module (which we call *PGCKA1*). Genome-wide pooled overexpression screens using a barcoded library of human open reading frames (ORFs) identified chr4p and other chromosomal regions that suppress proliferation when overexpressed in a context-dependent manner. Together, these results provide insight into TNBC, a difficult-to-treat cancer for which the current standard therapeutic options have not significantly changed the overall survival rate.

RESULTS

Loss of chromosome 4p is recurrent and functionally significant in basal breast cancer

We investigated the frequency of chromosome-arm losses in the breast cancer basal subtype.[8] The three most frequent chromosomal-arm losses in basal breast cancer were 8p, 5q, and 4p, occurring collectively in ~65%–76% of cases (Figures 1B and S1A; Table S1A). We detected chromosome 4p (chr4p) loss in ~65% of basal breast cancer cases, which has not been studied previously. Regions of chr4p loss span the entire short arm of chr4 without an apparent minimal deleted region, suggesting that its selective advantage is conferred by the loss of multiple genes residing within the chromosome arm rather than a single tumor-suppressor gene (Figure 1C).

Approximately 80% of genes (133 of 156) within chr4p showed reduced gene expression in patients with chr4p loss, indicating that this loss is functionally significant (Figure 1D and Table S1B). Among the ten genes with the most substantial reduction in gene expression upon chr4p deletion are *SLC34A2* and *RHOH*, which are known tumor-suppressor genes in other cancers but have not been previously implicated in breast cancer.[25] Notably, the worse survival of cases exhibiting chr4p loss is not due to the differences in the distribution of *TP53*

Figure 1. Loss of chromosome 4p in basal breast cancer is recurrent and functionally significant

(A) Experimental and analytic pipeline.

(B) The Cancer Genome Atlas (TCGA) invasive breast carcinoma single nucleotide polymorphism array dataset analysis shows the three most frequent chromosomal-arm losses in basal breast cancer.

(C) Regions of chr4p loss span a large fraction of the chromosome 4p. Segmented mean thresholds: dark blue, stringent threshold deletion < -0.3; light blue, lenient threshold $-0.3 <$ deletion < -0.1; light red, lenient threshold $0.1 <$ gain <0.3; red, stringent threshold gain >0.3; white, copy-neutral state.

(D) In TCGA basal breast cancer, ~80% of genes along chr4p decrease in expression due to chr4p loss.

(E) Overall survival of basal breast cancer patients with varying copy-number status of chr4p, $p < 0.0997$.

(F) Chr4p copy-number status across pan-cancer TCGA datasets.

(G and H) Gene set enrichment analysis showing representative terms that are enriched for genes displaying (G) elevated or (H) decreased expression due to chr4p loss in TCGA basal breast cancer.

loss-of-function mutations (Figure 1E and STAR Methods). Hence, chr4p loss may be clinically prognostic. We also detected statistically significant chr4p deletion in multiple cancer types, including lung squamous cell carcinoma, testicular germ cell tumors, and ovarian serous carcinoma, indicating that chr4p loss is broadly observed in other cancers (Figure 1F and Table S1C).

Next, we interrogated global transcriptomic changes associated with chr4p loss (Figures 1G and 1H; Table S1D). Basal breast cancers with chr4p loss showed an elevation of genes with roles in DNA replication ($p = 6.9 \times 10^{-7}$) such as *GINS4* encoding a member of the GINS complex, which plays an essential role in initiation of DNA replication and progression of DNA replication forks[26] and cell cycle ($p = 4.1 \times 10^{-4}$), such as *STK33* encoding a serine/threonine protein kinase, which activates the ERK signaling pathway.[27] Together, these terms suggest a proliferative advantage conferred by chr4p loss likely through a combined effect of expression changes of multiple genes belonging to these pathways. Elevated expression of genes with a role in microtubule cytoskeleton organization ($p = 1.1 \times 10^{-6}$) and protein translation ($p = 1.4 \times 10^{-3}$) suggest the involvement of cellular plasticity, which enables cancer cell adaptation to stress.[28] Chr4p loss was associated with decreased expression of genes annotated with positive regulation of immune response, cytokine-mediated signaling, T cell and B cell activation, interferon-γ-mediated signaling, and natural killer cell-mediated immunity consistent with TNBC displaying immune evasion and poorer outcome. This differential gene expression was not due to general differences in arm-level and chromosome-level copy-number changes ($p = 0.074$) (Figure S2A and Table S1E).[20] However, to be stringent we also corrected for aneuploidy by adding the aneuploidy score as a covariate in the differential gene expression analysis, which did not substantially change the enriched terms, with mitochondrial translation being the only term that became insignificant after the correction (Figures S2B and S2C). These findings support the suggestion that these global transcriptomic changes are specific to chr4p loss rather than general differences in aneuploidy between tumors and highlight the importance of specific chromosome-arm losses in basal breast cancer.

Chromosome 4p loss is an early event in basal breast cancer evolution

Evolution of basal breast cancer genomes can be reconstructed from somatic mutations detected by whole-genome sequencing (WGS).[17] To understand the evolutionary timing of chr4p loss, we performed phylogenetic reconstruction on bulk WGS of our primary tumor/patient-derived xenograft (PT/PDX) panel of 23 paired samples annotated to PAM50 basal breast cancer subtype for a total of 48 unique samples, which we previously collected.[29] To our knowledge, this is the largest available phylogeny for TNBC to date. Aggregated single-sample ordering revealed a typical timing of chromosome-arm aberrations and other genetic events (Figure 2A and Table S2A). Coding mutations in *TP53* had a high likelihood of being clonal and thus occurring early in tumor progression, consistent with it being a known driver event in basal breast cancer, as well as preceding whole-genome duplication consistent with *TP53* function and recent findings from single-cell, single-molecule DNA sequencing (DNA-seq) of eight human TNBCs and four cell lines.[18] We also observed that chromosomal-arm losses occurred before chromosomal gains, and chr17p loss harboring *TP53* occurred with timing similar to that of *TP53* coding mutations, likely driving biallelic inactivation of *TP53*. Most frequent and clonal chromosomal-arm losses included 4p, 17q, 3p, 17p, 15q, 14q, and 5q, the majority of which occurred after *TP53* coding mutations. Even though the median age of diagnosis for this PT/PDX cohort was 54, whole-genome duplication occurred ∼6 years prior, around 48 years of age in 16 samples (∼70%), and the most recent common ancestor was observed on average ∼2 years before diagnosis, around 52 years of age (Figure 2B and Table S2A). Unlike early events, there was no apparent subclonal structure since it tended to be distinct among patients, suggesting that even though early events are shared among basal breast cancer patients, late events diverge. These findings are consistent with a recent study on gastric cancer evolution, which showed that *TP53* leads to chromosome-arm-level aneuploidy in a temporally preferred order.[30] Thus, it is important to focus on clonal events for therapeutic application.

Since we detected *TP53* coding mutations occurring earlier than chr4p loss and no single known cancer gene was associated with chr4p loss, it is possible that a combination of cancer gene mutations in concert with *TP53* leads to a selective advantage of chr4p loss. To understand whether any of the early evolutionary events in our basal breast cancer cohort were already present in preinvasive cancer, we analyzed copy-number data of 95 patient samples diagnosed with ductal *in situ* carcinoma (DCIS) in a previously published study.[31] We found that *TP53* coding mutations and clonal losses of 3p, 4p, 5q, 8p, 13q, 14q, 16q, 17p, 17q, 21p, and 22q and gains of 1q, 8q, and 17q that were present in basal breast cancer were also detected in at least 20% of the DCIS samples, indicating that these chromosome-arm aberrations are key events in tumorigenesis (Figure S3A). To understand the selection pressures that maintain chr4p loss, we decided to focus on an individual patient (PT/PDX1735) whose trajectory revealed an early clonal chr4p loss, a clonal *TP53* coding mutation, and whole-genome doubling (Figure 2C), and for which we established a PDX and PDX-derived cell line. Our single-cell DNA-seq (scDNA-seq) analysis of PDX1735 confirmed that chr4p loss was an early event in this basal breast cancer progression (Figures S3B and S3C; Table S2B).

Chromosome 4p loss is associated with a proliferative state

To understand the functional effect of chr4p loss, we leveraged single-cell RNA sequencing (scRNA-seq) data from PDX1735, established from a basal breast cancer primary tumor, as reported in our previous study (Figure 3A, top left).[32] To infer copy-number status at single-cell resolution to identify transcriptional programs associated with cells harboring chr4p deletion (Figure 3), we employed a method that detects consistent variation in gene expression of consecutive genes across chromosomal regions.[33] To obtain a normal gene expression baseline, we performed scRNA-seq on breast tissue samples from two patients undergoing bilateral mammoplasty reduction

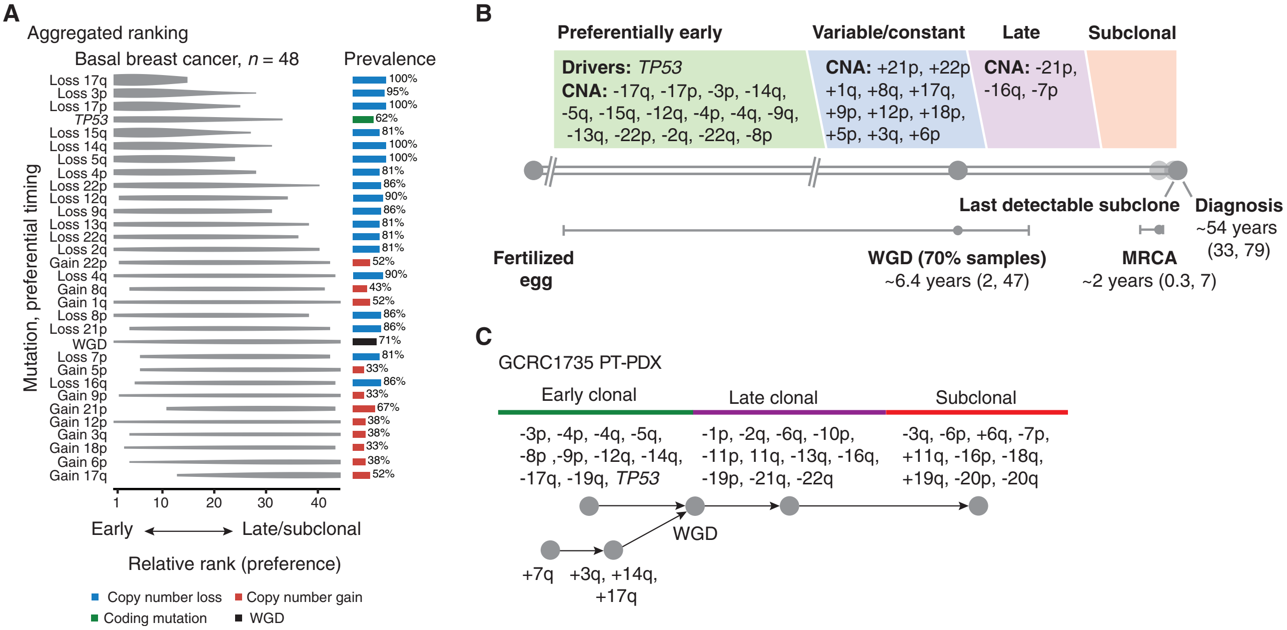

Figure 2. Chromosome 4p loss is an early event in basal breast cancer evolution

Basal breast cancer primary tumor/patient-derived xenograft (PT/PDX) panel was used for phylogenetic reconstruction.

(A) Aggregated single-sample ordering reveals typical timing of chromosome-arm aberrations. Preferential ordering diagrams show probability distributions revealing uncertainty of timing for specific events in the cohort. The prevalence of the event type in the cohort is displayed as a bar plot on the right. Losses occurring in >80% and gains occurring in >30% of all cases and evolutionary stages are depicted.

(B) Timeline representing the length of time, in years, between the fertilized egg and the median age of diagnosis for basal breast cancer. Real-time estimates for major events, such as whole-genome doubling (WGD) and the emergence of the most recent common ancestor (MRCA), are used to define early, variable, late, and subclonal stages of tumor evolution approximately in chronological time. Driver mutations and copy-number alterations (CNAs) are shown in each stage according to their preferential timing, as defined by relative ordering.

(C) An example of individual patient (PT/PDX1735) trajectory (partial ordering relationships).

(Figure S4 and Table S3A). We computed a Z score relative to the baseline and called copy-number aberrations using a hidden Markov model with three states: neutral copy number, loss, and gain. In this manner, we identified four stable "communities," groups of cells with a shared pattern of inferred copy-number profiles. Three communities (1–3) harbor chr4p deletion, and community 4 is enriched for cells with a chr4p copy-neutral state ($p = 0.002$; hypergeometric test) (Figure 3A, top right and bottom; Table S3B). The low number of cells (~5%) inferred to be copy neutral for chr4p (Figures S4C and S4D) is consistent with this loss being an early clonal event, as revealed by our timing analysis (Figures 2C and S3). This finding also agrees with our scDNA-seq analysis showing that chr4p copy-neutral cell frequency is ~2%, with the difference likely due to engraftment variability. The majority of cells also carry chr4q loss indicating a loss of the entire chr4. The low frequency (~3.5%) of chr4q copy-neutral cells is consistent with it being an early clonal event, as confirmed by our timing analysis based on bulk WGS (Figures 2C, S3B, and S3C). It is possible that chr4p loss preceded chr4q loss, which is suggested by our timing analysis on the entire panel of primary tumors and PDXs (Figures 2A and 2B), and future work with higher sequencing coverage would be needed to further refine the early evolutionary epoch into finer evolutionary stages to understand the branching points.

To understand the selection pressures that maintain chr4p loss, we compared the distributions of chr4p copy-neutral and deletion communities across different cellular clusters associated with distinct transcriptional programs. We previously described six cellular clusters embedded within PDX1735, which included proliferation, nuclear/mitochondria, antigen presentation, basal, mesenchymal/stem, and boundary, based on differential gene expression.[32] As expected, the inferred copy-number communities did not overlap with any specific gene expression cluster, since the normalized expression was smoothed using a rolling median approach to reduce the effect of single-gene outliers (Figure S4B). Thus, cells belonging to chr4p deletion communities (communities 1 + 2 + 3), which comprised most cells of the PDX1735, did not show a preferential distribution across any cellular cluster (Figure 3B). However, cells belonging to the chr4p copy-neutral community (community 4) were significantly strongly depleted in the proliferation and to a lesser extent in the mesenchymal cluster (Figure 3B). The proliferation cluster was characterized by proliferative cells, since a large proportion of cells in this cluster (~95%) were cycling and exhibited inferred G_2/M and S cell-cycle states based on the relative gene expression of G_1/S and G_2/M gene sets[34] as well a high expression of cell-cycle genes, such as *MKI67* (Figure 3C and Table S3B).

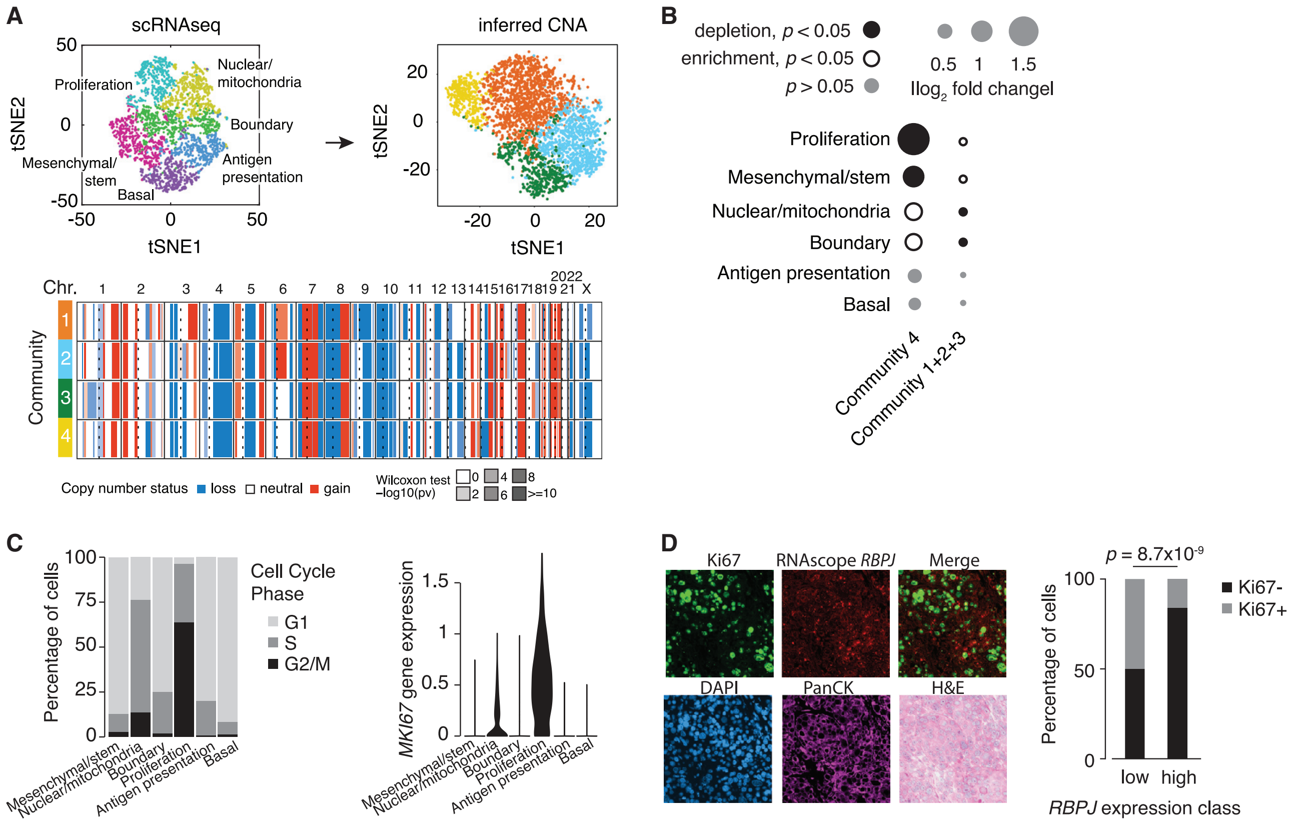

Figure 3. Chromosome 4p loss is associated with a proliferative state

(A) Single-cell RNA-sequencing data of PDX1735 (top left) from a previous study[32] were used to infer copy-number status (top right) and displayed using t-distributed stochastic neighbor-embedding plots, colored by shared gene expression or inferred copy-number profiles. Heatmap (bottom) shows the inferred copy-number profile of three communities harboring chr4p deletion (1–3) and community 4 enriched for cells with chr4p copy-neutral state. chr denotes chromosome, dashed line denotes centromere, and solid line denotes start/end of chromosome. Loss is blue, copy neutral is white, and gain is red. Likelihood of inferred copy-number change is represented by Wilcoxon test $-\log_{10}$ p value.

(B) Frequency of cells inferred to harbor chromosome 4p deletion or copy-neutral state across “transcriptional program” clusters. The size of the circle reflects the fold increase over the background fraction of all cells in a specific gene expression cluster. Significance was assessed by a hypergeometric test; $p < 0.05$. Solid black circles, depletion; open black circles, enrichment; gray, no change.

(C) Distribution of cells across (left) cell-cycle phases (light gray, G_1 phase; medium gray, S phase; black, G_2/M phase) and (right) *MKI67* gene expression using “transcriptional program” clusters from (A).

(D) Staining of paraffin-embedded fixed tissue section of PDX1735 using a combination of immunofluorescence (IF) for Ki67 (marker of proliferation), RNA *in situ* hybridization (ISH) for *RBPJ* (marker of chr4p), DAPI staining for nuclei, pan-cytokeratin (PanCK) IF for epithelial cancer cells, and H&E staining for cancer histology. Significance was assessed by Fisher’s exact test.

To functionally validate the findings from scRNA-seq data, we performed immunofluorescence staining combined with RNA *in situ* hybridization (RNA ISH). The staining of a paraffin-embedded fixed tissue section of PDX1735 relied on a combination of immunofluorescence for Ki67 and RNA ISH for *RBPJ*. *RBPJ* was selected as a marker of chr4p copy-number state because of the availability of a probe for RNA ISH and a consistent gene expression difference in our basal breast cancer PT/PDX panel between chr4p copy-neutral and deletion samples. This analysis revealed that there was an inverse relationship between Ki67 abundance and *RBPJ* gene expression. Breast cancer cells with chr4p deletion and thus low *RPBJ* expression showed a high abundance of Ki67 and thus were more proliferative than chr4p copy-neutral cells (Fisher’s exact test, $p = 8.7 \times 10^{-9}$) (Figure 3D). This is consistent with the transcriptomic changes detected in basal breast cancer patients due to chr4p loss, which showed an upregulation of expression of genes important for proliferation by being involved in processes such as DNA replication, cell cycle, and translation and downregulation of expression of genes involved in apoptosis (Figures 1G and 1H). Together, these findings suggest that chr4p loss confers on basal breast cancer cells a proliferative advantage.

Suppression of proliferation by overexpression of chromosome 4p genes is context dependent

To determine whether chr4p deletion in basal breast cancer is selected as a result of proliferative advantage, we tested whether the overexpression of genes within this region elicits a proliferation defect. chr4p copy-neutral normal breast epithelial cell

CellPress
OPEN ACCESS

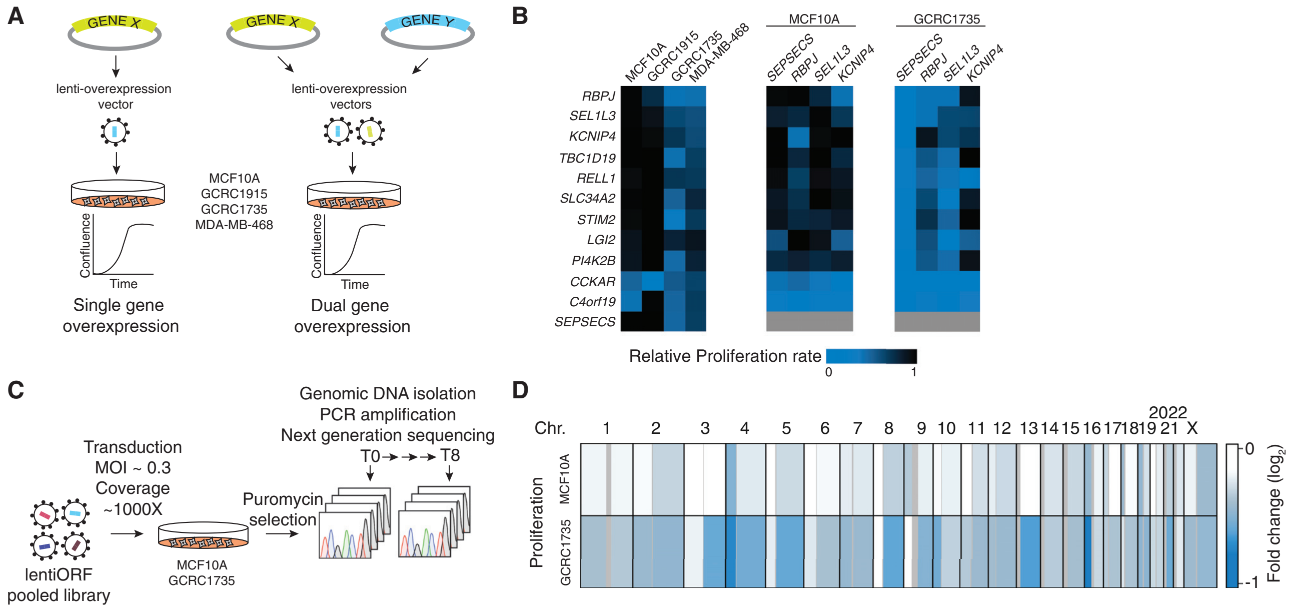

Figure 4. Overexpression of chromosome 4p genes leads to context-dependent suppression of proliferation

(A) Schematic of gene overexpression strategy in chr4p copy-neutral cells MCF10A, GCRC1915, or chr4p deletion cells GCRC1735, MDA-MB-468.

(B) Single or dual chr4p gene overexpression confers a proliferation defect in chr4p loss but not in chr4p copy-neutral cell lines. Blue, proliferation defect; black, no change relative to control. n = 3 with three technical replicates.

(C) Schematic of the genome-wide pooled LentiORF overexpression screen.

(D) Genome-wide pooled LentiORF overexpression screen revealed genomic regions with context-dependent suppression of proliferation, e.g., chr4p and 13q. Blue, proliferation defect; black, no change relative to T0 control.

line, MCF10A, chr4p copy-neutral basal breast cancer PDX-derived cell line, GCRC1915, and chr4p deletion basal breast cancer cell line MDA-MB-468 and a PDX-derived counterpart, GCRC1735, were used to generate stable cell populations overexpressing candidate chr4p genes using lentivirus-mediated integration of constructs from the human ORF collection (Figure 4A).[35] The candidate genes resided within a high-confidence chr4p deletion region in GCRC1735 according to whole-exome sequencing data from our previous study,[32] encompassing about half of the chromosome arm and containing 30 genes, for about half of which our arrayed collection contained LentiORF overexpression vectors. These vectors were individually validated by a combination of restriction digest and Sanger sequencing (see STAR Methods).

Surprisingly, overexpressing a large fraction of chr4p genes suppressed proliferation in a context-dependent manner. Thus, proliferation suppression was only observed in basal breast cancer cell lines that were deleted for chr4p, GCRC1735 and MDA-MB-468, and not in cell lines that were chr4p copy neutral, MCF10A or GCRC1915. GCRC1915 displays loss of heterozygosity within chr4p making it copy neutral with no change in chr4 copy number (Figure 4B, left). This extent of suppression was further exacerbated when two random genes within chr4p were overexpressed (Figure 4B, right). The context dependency of suppression of proliferation of chr4p genes is likely not due to *TP53* mutation status, since GCRC1915, GCRC1735, and MDA-MB468 harbor a coding mutation in *TP53*. On the other hand, GCRC1735, unlike GCRC1915, harbors a germline *BRCA1* mutation,[29] clonal losses chr1p, chr19p, chr19q, chr21q, and chr22q, and clonal gains chr3q, chr7q, chr14q, and chr17q (Table S2A), which may underlie the context-dependent suppression of proliferation of chr4p gene overexpression observed in GCCR1735 and not in GCRC1915. It was recently reported that MDA-MB-468 also carries a deletion of exon 12 in *BRCA2* that impairs homologous repair[36] and may contribute to phenotype similar to that of GCRC1735. Since both chr4p and chr22q losses are often early clonal events in basal breast cancer (Figure 2B), it is possible that their interaction rewires chr4p, maintaining it in a deletion state by selecting against chr4p amplification. Hence, the observed context-dependent suppression of proliferation may be due to a genetic interaction with another genetic aberration, which rewires the genetic network, sensitizing chr4p region to overexpression and thus maintaining it in a deletion state. Further studies in isogenic model systems should be conducted to test which combinations of genetic events interact with chr4p loss to confer a proliferative advantage, since it was previously found that individual chromosome-arm losses lead to growth defects.[24]

To determine whether the context-dependent suppression of proliferation was specific to chr4p and to identify other such regions, we conducted a genome-wide overexpression screen using the pooled TRC3 LentiORF collection as previously described (Figure 4C and Table S4).[37] Two cell lines with different copy-number states of chr4p, MCF10A (chr4p copy neutral), and GCRC1735 (chr4p deletion) were transduced with the pooled LentiORF library at multiplicity of infection of 0.3 to ensure one integration event per cell. After

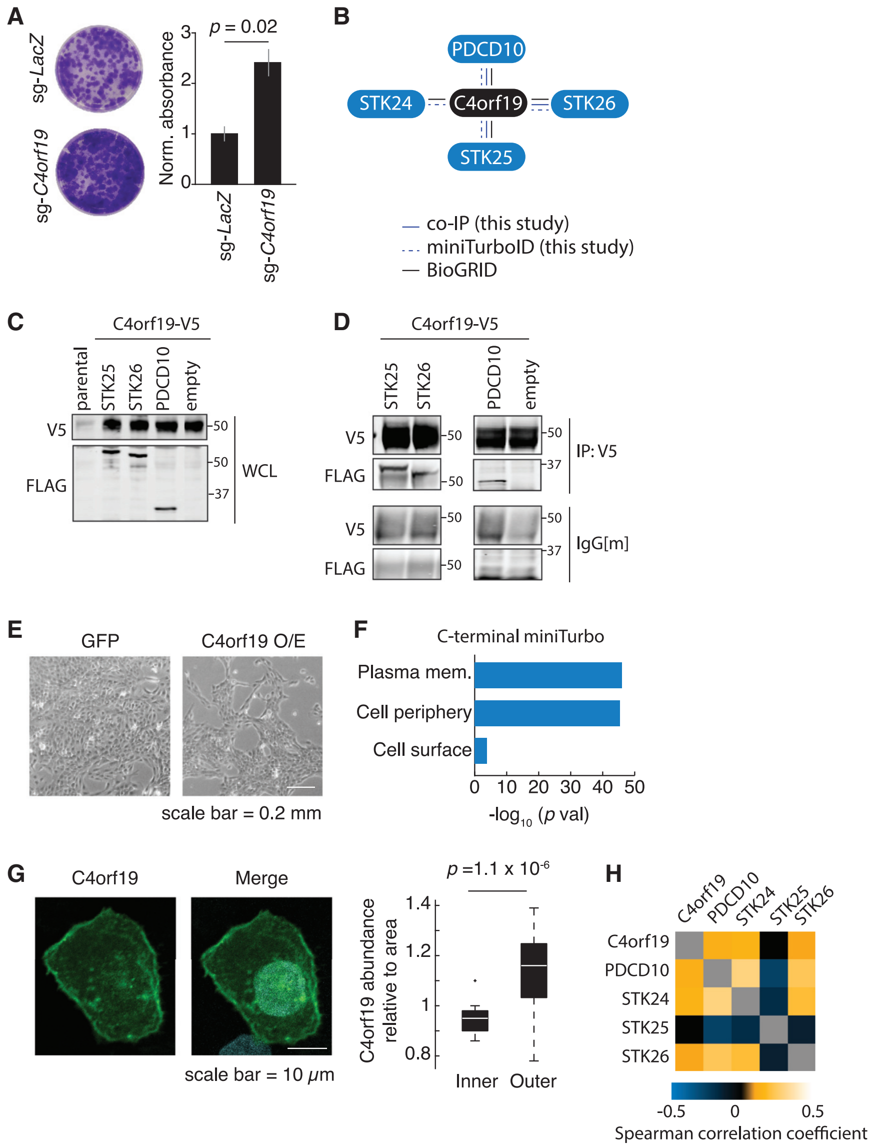

Figure 5. C4orf19 (PGCKA1) is associated with the PDCD10-GCKIII module

(A) Colony-formation assay for MCF10A cells treated with a control sgRNA-*LacZ* and sgRNA-*C4orf19*. A representative well per condition is shown and quantified. Significance was assessed using a Wilcoxon rank-sum test. Data are presented as mean ± SD, n = 3, two technical replicates.

(B) Summary of coIP assay results from (D), miniTurbo ID conducted in MCF10A cells expressing C4rf19-miniTurbo from (F), and literature curation using BioGRID.

(C) Western blot using whole-cell lysate shows heterologous expression of C4orf19-v5 and PDCD10-GCKIII module 3×FLAG-PDCD10/STK25/STK26 in MCF10A cells. n = 3.

(D) CoIP assay using Anti-V5 for pull-down shows the presence of 3×FLAG-PDCD10/STK25/STK26 in MCF10A cells. n = 3.

(E) Representative bright-field microscopy images of MCF10A cells overexpressing GFP control or C4orf19 48 h post-induction with doxycycline. n = 3.

(F) Analysis of gene ontology molecular function of proteins in proximity to C4orf19 from miniTurbo ID from (C) shows enrichment of proteins at the plasma membrane.

(G) Immunofluorescence of C4orf19 indicates its localization at the cell periphery and quantification using n = 3 biological replicates comprising 27 cells. Significance was assessed using a Wilcoxon rank-sum test.

(H) Heatmap shows co-expression for the members of the C4orf19-PDCD10-GCKIII module using the Cancer Cell Line Encyclopedia mRNA expression dataset; Spearman correlation coefficient, $p < 0.05$ (see STAR Methods). Gray indicates missing data.

puromycin selection, cells were maintained for 6–8 doublings, and 1,000× coverage was maintained at each step of the experiment. Next-generation sequencing was used to capture barcode abundance, which served as a proxy for cell-growth rate. A comparison with a previously reported genome-wide pooled LentiORF overexpression screen in a normal human mammary epithelial cell line uncovered that ~67% of genes that were identified as STOP genes were also scored as suppressing proliferation in MCF10A in this study, such as epithelial tumor suppressor *ELF3*, transcription factor *EBF1*, and a DNA repair protein *RAD51*.[19] The screen also revealed regions that suppressed proliferation in a context-dependent manner (Figure 4D). The context-dependent regions that suppressed proliferation when overexpressed in GCRC1735, but not in MCF10A, included chr4p and 13q. These regions were also deleted in GCRC1735 and not in MCF10A, suggesting that this mode of selection is not specific to chr4p loss and likely exerts the selection pressure early in tumor progression, since our evolutionary timing analysis revealed that both are clonal events (Figure 2C). These observations suggest that the dosage of these genes exerts a selection pressure to maintain this chromosomal region deleted in a specific genomic context of basal breast cancer.

Overexpression of *C4orf19* suppresses proliferation and reveals an interaction with PDCD10-GCKIII kinase module

We observed that *C4orf19* suppressed proliferation when overexpressed in multiple contexts, such as MCF10A, GCRC1735, and MDA-MB-468 cells, indicating its tumor-suppressive role (Figure 4B). To test this directly, we used CRISPR-Cas9 to generate a loss of function of *C4orf19* in MCF10A, which resulted in a proliferative advantage compared to control cells, consistent with it being a tumor-suppressor gene within chr4p (Figures 5A and S5A; STAR Methods). Thus, despite a widespread context dependency of the suppression of proliferation, there were certain genes that suppressed proliferation independently of context,

representing more general tumor-suppressor genes. To improve the understanding of the human genome more broadly and the functional role of chr4p more specifically, we conducted further analyses to understand the biological role of *C4orf19*. C4orf19 is an uncharacterized protein 314 amino acids in length, which has orthologs in mouse and rat according to the Alliance of Genome Resources ortholog inference.[38] Functional analysis of its protein sequence using InterPro[39] revealed that it belongs to the protein family domain unknown function DUF4699 and is predicted to contain two consensus disorder regions (36–142, 267–291). The disordered protein structure encoded by *C4orf19* was also supported using the AlphaFold predicted model.[40] Next, we mined BioGRID[41] for previously identified protein-protein interactions: high-throughput methods, such as affinity capture-mass spectrometry (MS) and yeast two-hybrid, both revealed PDCD10 (Figure 5B).[42,43] Additionally, all three members of the germinal center kinases (GCKIII) subfamily, STK24, STK25, and STK26, which directly interact with PDCD10 in a mutually exclusive heterodimer,[44] were reported in the affinity capture-MS method.[43] We heterologously expressed C4orf19-V5 and 3×FLAG-PDCD10, 3×FLAG-STK25, and 3×FLAG-STK26 in MCF10A cells (Figure 5C). Co-immunoprecipitation (coIP) assay using Anti-V5 for pull-down showed the presence of 3×FLAG-PDCD10, 3×FLAG-STK25, and 3×FLAG-STK26, confirming previously identified high-throughput interactions, indicating that C4orf19 interacts with PDCD10 and its associated GCKIII kinases (Figure 5D).

To gain more insight into the biological role of C4orf19, we performed proximity-dependent biotinylation of proteins coupled to MS (miniTurboID)[45] to reveal the comprehensive physical neighborhood in which C4orf19 resides. MiniTurbo biotin ligase was fused to C4orf19 and expressed in MCF10A (alongside negative controls; bait expression was verified by western blotting), and biotinylation of proximal proteins was induced by the addition of biotin (Figures S5B and S5C). Biotinylated proteins were recovered by streptavidin-affinity chromatography and identified by MS. Reduction in proliferation was observed 48 h after induction of *C4orf19* overexpression with doxycycline (Figure 5E). Using the SAINTexpress computational tool, we identified 370 high-confidence (Bayesian false discovery rate <5%) proximal interactors, which included PDCD10, STK24, STK25, and STK26 (Figure 5B and Table S5). The analysis of gene ontology (GO) molecular function of C4orf19 proximal interactors showed enrichment of proteins at the plasma membrane, suggesting that C4orf19 is localized to the cell periphery (Figure 5F). The subcellular localization of C4orf19 at the cell periphery was further validated by immunofluorescence of C4orf19. The signal intensity of C4orf19 relative to area of the inner or outer cell section indicates that C4orf19 abundance is higher at the cell periphery, although it was not uniformly distributed (Figure 5G). This finding is consistent with the Human Protein Atlas, which showed that across tissues C4orf19 is at the cell periphery in some cancers, such as breast lobular carcinoma and RT4 cells derived from a urinary bladder transitional cell papilloma.[46,47] Similarly, the Alliance of Genome Resources computationally predicted its localization to cell junctions based on GO annotation and orthology.[38] We did not observe C4orf19 in the nucleus, which is provided as a secondary predicted localization of this protein, perhaps due to cell-type specificity.

These findings support the notion that C4orf19 is associated with a subset of PDCD10 and GCKIII kinases localized to the cell periphery, and we propose to rename *C4orf19* as *PGCKA1* (PDCD10-GCKIII kinases associated 1). *STK24* resides on chr13q and *STK25* on chr2q, both of which show a deletion state in GCRC1735 and suppress proliferation when overexpressed (Figures 2C and 4D), suggesting copy-number change as a selection for stoichiometric balance of the members of the PDCD10-GCKIII kinase module. Overall, the deletions of chr13q and chr4p are both early clonal events in the basal PT/PDX panel, highlighting the potential importance of the stoichiometric balance of the PDCD10-GCKIII kinase module as an additional common evolutionary mechanism in this breast cancer subtype. To understand whether this finding is also conserved across other cancer types, we used the entire Cancer Cell Line Encyclopedia mRNA expression dataset to obtain the co-expression of all the pairwise combinations of C4orf19, PDCD10, STK24, STK25, and STK26. There was significant and positive correlation for all pairwise comparisons with the exception of STK25, which showed a negative correlation (Figure 5H). This analysis suggests that the members of the C4orf19-PDCD10-GCKIII module are co-regulated across a range of cancer types and, thus, that the stoichiometry of this protein module is important across multiple cancers. The negative correlation of all the members with STK25 suggests that it is regulated differently but that its stoichiometric balance within the module is still important.

DISCUSSION

This study identified chr4p loss as a frequently recurrent chromosome-arm loss affecting a large fraction of basal breast cancer patients. Our data indicate that chr4p loss is functionally significant and evolves early in tumorigenesis. Through multiple approaches, we showed that the deletion of chr4p is associated with enhanced proliferation. Targeted single and dual-gene overexpression assays of genes within chr4p uncovered *C4orf19*, which suppressed proliferation and was identified to be associated with the PDCD10-GCKIII kinase module, and we propose to rename this gene *PGCKA1*. However, most genes within chr4p suppressed proliferation when overexpressed in a context-dependent manner associated with chr4p deletion. The growth defect observed to arise from chr4p gene overexpression is not due to general hypersensitivity to overexpression of GCRC1735 and MDA-MB-468 because all the data were normalized to the growth of control cells overexpressing GFP. Genome-wide pooled overexpression screens identified other chromosome arms whose suppression of proliferation was context dependent and associated with copy-number loss. Together, these findings enhance our understanding of the early emergence of complex aneuploid karyotypes involving chr4p in breast cancer.

We found that chromosome-arm losses are hemizygous, likely due to the presence of core essential genes residing within them (Figure S1A).[48,49] The apparent lack of a clear minimal deletion region of chr4p in basal breast cancer suggests that this event is not driven by the loss of a single tumor-suppressor gene but rather by multiple genes and/or genetic elements at multiple,

spatially separated loci whose deletion together yields a proliferative advantage. While there were four previously known tumor-suppressor genes within chr4p, none of them has been implicated in breast cancer.[25] These include *SLC34A2*, which encodes a pH-sensitive sodium-dependent phosphate transporter[50] and *N4BP2*, which encodes 5′-polynucleotide kinase, playing a role in DNA repair, both of which have been implicated in lung cancer.[51] *RHOH* encodes a member of the Ras superfamily of guanosine triphosphate (GTP)-metabolizing enzymes and has been implicated in non-Hodgkin's lymphoma.[52] *PHOX2B* is a transcription factor involved in neuroblastoma.[53] In contrast to *PHOX2B*, which is recessive, the tumor-suppressive effects of *SLC34A2* and *RHOH* are dominant,[25] indicating that the perturbation of one copy of these genes is sufficient to contribute to carcinogenesis, and their combined effect due to chr4p loss may be providing a selective advantage in TNBC. Surprisingly, the expression of several genes is elevated, likely due to the loss of *cis*-transcriptional repressors. These include *UCLH1*, which is a ubiquitin hydrolase previously shown to be highly expressed in metastatic estrogen-receptor-negative breast cancer and THBC subtypes.[54]

Simultaneous gene expression silencing using RNAi of multiple combinations of up to three genes along chr8p inhibited tumorigenesis in a mouse model of hepatocellular carcinoma, indicating that multiple tumor-suppressor genes show a greater capacity to promote tumorigenesis than individual genes.[55] The effect of chr3p loss in lung cancer is also attributed to an alteration in a combination of genes.[24,56] This is similar to the selection for chr5q loss in TNBC, because it was previously established in a murine model that 5q loss of heterozygosity leads to a loss of function of *KIBRA*, which encodes a multi-domain scaffold protein that inhibits oncogenic transcriptional co-activators YAP/TAZ that mediate mechanotransduction signals.[21] Chr5q also harbors *RAD17*, *RAD50*, and *RAP80* genes that are important for *BRCA1*-dependent DNA repair, and their loss impairs *BRCA1*-pathway function critical for DNA-damage control, contributing to increased genomic instability.[57] In addition, since noncoding genes including long noncoding RNA and microRNA reside within chr4p, it is possible that their loss leads to overexpression of certain target genes contributing to a proliferation state. The apparent lack of a minimal region in a large chromosomal aberration has been also previously observed in human embryonic stem cells and induced pluripotent stems cells when screening for genetic changes occurring in cell culture to evaluate their tumorigenicity, which reported a recurrent gain of chromosomes 1, 12, and 17 without any frequently repetitive minimal amplicon.[58]

The functional significance of chr4p loss was assessed by the reduction in gene expression of a large portion of genes within the region as well as the association of the chromosome-arm loss with a proliferative state and mesenchymal state. This is consistent with previous observations that hallmark sets of cell cycle and epithelial-mesenchymal transition genes were upregulated when chr3p was lost.[24] Chr4p loss in basal breast cancer was also associated with a decrease in immune signature, suggesting that specific partial aneuploidy of chr4p rather than general differences in aneuploidy between tumors may be important for immune system evasion, which were previously reported in a pan-cancer analysis.[20] This is especially important, since it was previously shown that TNBC with an "immune-cold" microenvironment characterized by the absence of $CD8^+$ T cells in the tumor resulted in poor outcomes.[59] The finding of chromosome-arm-specific aneuploidy exerting distinct effects on the immune system is likely due to the variation in the composition of immune-related genes. This is consistent with a finding that chr3p loss is associated with increased immune activity.[24] Furthermore, despite chr4p loss being prevalent in the *HER2* amplified group, it is not associated with decreased survival (Figure S1B). This finding likely indicates that *HER2* amplification, a key event in these tumors, masks the effect whereby the double mutant carrying both chr4p loss and *HER2* amplification resembles the more extreme phenotype of the single mutant *HER2* rather than the combined effect of both aberrations.

Timing analysis showed a preferred order for chromosome-arm-level copy-number allteration in basal breast cancer. This is consistent with the evolution of genetically engineered gastric cancer organoids.[30] In fact, the evolutionary early alterations in similar chromosome arms, such as the loss of chr3p, chr4p, and chr4q, were detected in both studies, suggesting convergent mechanisms for regulating tumor emergence in gastric and breast cancer, which may be applicable to other cancers. The finding that chr4p loss along with other early evolutionary events in breast cancer are already detected in DCIS, which is the most common form of preinvasive breast cancer, indicates the key role played by these chromosomal aberrations in tumor initiation and progression. It has been previously proposed that the cancer genome is shaped by sensitivity to a change in gene dosage of tumor-suppressor genes and oncogenes caused by chromosome-arm loss or gain.[60] Our observation of context-dependent suppression of proliferation of a frequently lost chromosome arm suggests that additional mechanisms exist that maintain cancer cells with specific chromosome-arm losses. A recent pan-cancer evolutionary study noted recurrent early genetic events and the broadening of this set in later stages, suggesting a preference for these genomic changes in early tumor evolutionary stages and potential genetic interactions that constrain the evolution.[17] This is consistent with previous studies suggesting that despite aneuploidy resulting in a growth disadvantage due to proteotoxic and metabolic stress, it may lead to increased selective pressure on cells to acquire growth-promoting genetic alterations.[24,61] Since co-occurring aneuploidies are often observed in stem cell cultures, tumors, and yeast cells, genetic interactions between aneuploidies have been recently suggested to be involved in cancer genome evolution.[62–64]

Our study revealed that PGCKA1 (C4orf19) is physically associated with PDCD10 and a subfamily of GCKIII: STK24, STK25, and STK26. It is possible that PGCKA1 is a direct binder of PDCD10 because the likelihood of an interaction between two human proteins in a yeast two-hybrid assay when at least one is not nuclear, PDCD10, is very high to be direct.[42] PGCKA1 is likely excluded from the striatin-interacting phosphatase and kinase (STRIPAK) complex, a large multi-protein assembly, which was initially characterized in HEK293 cells.[65] This is supported by the lack of evidence of striatins in the PGCKA1 miniTurboID data or other publicly available protein-protein interaction data.

This finding further supports the direct binding of PGCKA1 to PDCD10 and suggests that PGCKA1 competes with striatins for binding to PDCD10. The interface that is mediating this interaction between PGCKA1 and PDCD10 may thus be the same as the interface required for binding of striatins to PDCD10 or other partners, which was previously shown to be occurring in a mutually exclusive manner.[44,66] Thus, we propose that PGCKA1 is a protein that can tether the GCKIII kinases through the PDCD10 adapter to the plasma membrane bridging the GCKIII kinases to a substrate in this locale.

PDCD10 is also known as *CCM3*, a causative gene of cerebral cavernous malformation, a neurovascular disease characterized by vascular malformations.[67] In addition to interacting with and controlling signals emanating from the CCM2/CCM1 pathway in cytoskeletal organization, PDCD10 also regulates STRIPAK and potentially other pathways also implicated in vascular integrity.[67] PDCD10 is also pro-apoptotic and controls the cell cycle, entry into senescence, apoptotic response to oxidative damage, inflammation, and DNA-damage repair as well as cell migration.[67] Its many functions are thought to be enabled by its multiple subcellular localizations, including cell-to-cell junctions and the Golgi apparatus. Our findings suggest that since PGCKA1 is at the cell periphery, it likely interacts with PDCD10 at the cell-to-cell junctions. PDCD10 heterodimerizes with GCKIII kinase subfamily and modulates cell migration by regulating Golgi assembly which is mediated by its interaction with STK25,[68] regulates exocytosis through its interaction with STK24 which when lost results in oxidative damage and dismantling of the adherens junctions,[69] and maintains ion homeostasis through its interaction with STK26.[70] Thus, PGCKA1, through its interaction with PDCD10, may tether GCKIII STK24, STK25, and STK26 to the membrane. This could affect diverse processes that decrease cell proliferation when *PGCKA1* is overexpressed and contribute to the proliferation and mesenchymal transcriptomic signatures observed in cell populations with chr4p loss. The similarity of the context-dependent suppression of proliferation of chr4p genes to chr13q, a region deleted in ~45% of basal breast cancers, which also harbors a PDCD10 heterodimerization partner, STK24, highlights the important role of PDCD10-kinase module stoichiometric balance in exerting selection pressures on copy-number evolution of the breast cancer genome.

The suppression of proliferation of chr4p genes when overexpressed suggests a mechanism of negative selection of chr4p gain in cancers. We showed that chr4p loss was broadly observed across multiple cancer types. A recent pan-cancer analysis of cancer aneuploidy similarly detected chr4p loss in squamous, gynecologic, and gastrointestinal tumors.[24] Since chr4p loss contains multiple genes that promote tumorigenesis when co-deleted, their simultaneous loss may result in vulnerabilities that cannot be identified by studying single genes and thus could provide potential therapeutic avenues for patients with TNBC and other cancers.

Limitations of the study

The analysis of the branching points within the early evolutionary epoch is limited by sequencing coverage, and future work with higher sequencing coverage would be needed to reveal finer evolutionary stages. The screening results are limited by the completeness of the LentiORF library. In the future, an updated LentiORF library with a larger set of constructs per gene covering a larger extent of the genome would provide more robust conclusions about the context specificity of suppression of proliferation due to chr4p gene overexpression. This study is also limited by a relatively small number of cell lines that may not generalize to all chr4p loss basal breast cancer cell lines or to normal breast epithelial cell lines of different genetic backgrounds. In the future, conducting a differential overexpression analysis by screening more karyotypically normal and genomically aberrant cancer cell lines representing diverse genetic backgrounds and originating from different tissues would be necessary. This would lend further support to the conclusion that there is genetic network rewiring in cancers that drives the selection of specific chromosomal abnormalities. The screen results in cancer cell lines with a diverse spectrum of genomic aberration confound the effects of individual chromosome-arm gains and losses. Constructing an isogenic panel of mutant cell lines to isolate the effect of single chromosome-arm aberrations as well as their combinations would be instrumental for future studies.

STAR★METHODS

Detailed methods are provided in the online version of this paper and include the following:

- KEY RESOURCES TABLE
- RESOURCE AVAILABILITY
  - Lead contact
  - Materials availability
  - Data and code availability
- EXPERIMENTAL MODEL AND STUDY PARTICIPANT DETAILS
  - Isolation of normal breast epithelial single Cell suspension
  - Cell culture
- METHOD DETAILS
  - Single and dual-gene overexpression assay
  - Genome-wide pooled overexpression screen
  - TCGA data computational analysis
  - Immunoprecipitation and western blot
  - miniTurboID
  - RNA *in situ* hybridization and immunofluorescence of GCRC1735
  - Colony formation assay of *C4orf19* loss-of-function CRISPR-Cas9 mutant cells
  - C4orf19 immunofluorescence and quantification
  - Coexpression of the C4orf19-PDCD10-GCKIII module
  - Single cell RNA sequencing
  - Single-cell RNA sequencing analysis
  - Inferring copy number aberrations from scRNA-seq
  - WGS analysis
  - Timing analysis using bulk WGS data for basal breast cancer PT/PDX panel
  - scDNA-seq
  - scDNA-seq data processing
  - scDNA-seq SNV analysis

- ○ Building the clone tree and assigning SNVs to clones using heuristics
- ○ Inference of haplotype-specific copy number profiles in single cells using CHISEL
- ○ Inferring the most recent common ancestor (MRCA) copy number profile
- ● QUANTIFICATION AND STATISTICAL ANALYSIS

SUPPLEMENTAL INFORMATION

Supplemental information can be found online at https://doi.org/10.1016/j.celrep.2024.113988.

ACKNOWLEDGMENTS

We thank Anie Monast and Virginie Pilon for assistance with animal experiments, Valentina Munoz Ramos for lab organization and management, Margarita Souleimanova and Nicholas Bertos for handling mammoplasty reduction surgery tissue, and Chris Law and the Center for Microscopy and Cellular Imaging at Concordia University for guidance and help with the image analysis. Single-cell RNA and DNA sequencing library preparation and sequencing were performed by Yu Chang Wang at the McGill Genome Center, and confocal microscopy was performed at the Center for Microscopy and Cell Imaging, Concordia University and Advanced BioImaging Facility, McGill University. Histology sections were prepared and imaged at the Histology Innovation Platform, lentiviral ORF collections were obtained from the McGill Platform for Cell Perturbation, and flow cytometry was conducted at the Flow Cytometry Core Facility, Goodman Cancer Institute. Proteomics was performed at the Network Biology Collaborative Center at the Lunenfeld-Tanenbaum Research Institute (supported by the Canada Foundation for Innovation, the Ontario Government, Genome Canada, and Ontario Genomics [OGI-139]). The breast tissue bank at McGill University (from which PDXs and cell lines were generated) is supported by the Réseau de Recherche en Cancer of the Fonds de Recherche du Québec - Santé and the Quebec Breast Cancer Foundation (to M.P.). This work was supported by the Cancer Research Society (M.P. and E.K.), the Canadian Institutes of Health Research (FDN 143301 to A.-C.G., FDN 143281 to M.P., and PG 480465 to E.K.), Canada Research Chair (CRC-2021-00031 to E.K.), the Terry Fox Research Institute (to A.-C.G.), Genome Canada Genomic Platform grants (CFI 33406 and 40104 to J.R.; CFI MSI 35444 to J.R.), Canada Foundation for Innovation (CFI 41563 to E.K.), and Worldwide Cancer Research (16-0402 to M.P.). T.M.B., T.L., and P.V.L. were supported by the Francis Crick Institute, which receives its core funding from Cancer Research UK (CC2008), the UK Medical Research Council (CC2008), and the Wellcome Trust (CC2008). For the purpose of Open Access, the authors have applied a CC BY public copyright license to any Author Accepted Manuscript version arising from this submission. P.V.L. is a Winton Group Leader in recognition of the Winton Charitable Foundation's support toward the establishment of The Francis Crick Institute. P.V.L. is a CPRIT Scholar in Cancer Research and acknowledges CPRIT grant support (RR210006). Additional support was provided by Fonds de Recherche du Quebec-Sante Postdoctoral Fellowship to E.K., L'Oreal Canada Women in Science Research Excellence Fellowship to E.K., Canadian Institutes of Health Research Banting Postdoctoral Fellowship to E.K., Cancer Research Society Scholarship for the Next Generation of Scientists Postdoctoral Fellowship to E.K., and CIHR Doctoral Fellowship to K.T.A. T.M.B. is supported by a PhD fellowship from Boehringer Ingelheim Fonds.

AUTHOR CONTRIBUTIONS

Conceptualization, E.K. and M.P.; methodology and investigation, E.K., K.T.A., P.P.C., M.S., J.D.C., D.Z., G.M., H.K., A.-M.F., and J.R.; formal analysis, E.K., T.M.B., T.L., J.M., K.T.A., J.D.C., G.M., A.P., Y.Y., J.B., R.L., M.B., M.-C.G., A.O., Q.M., C.L.K., S.H., A.-C.G., J.R., G.B., P.V.L., and M.P.; resources, C.M. and P.G.D.; writing – original draft, E.K. and M.P.; writing – review & editing, E.K., T.M.B., T.L., J.M., K.T.A., P.P.C., C.M., J.B., H.K., R.L., M.B., A.-M.F., A.O., Q.M., C.L.K., S.H., A.-C.G., J.R., G.B., P.V.L., and M.P.; supervision, M.P.; funding acquisition: A.-C.G., J.R., P.V.L., and M.P.

DECLARATION OF INTERESTS

The authors declare no competing interests.

Received: September 1, 2023
Revised: February 2, 2024
Accepted: March 7, 2024
Published: March 22, 2024

REFERENCES

1. Hammond, M.E.H., Hayes, D.F., Dowsett, M., Allred, D.C., Hagerty, K.L., Badve, S., Fitzgibbons, P.L., Francis, G., Goldstein, N.S., Hayes, M., et al. (2010). American Society of Clinical Oncology/College Of American Pathologists guideline recommendations for immunohistochemical testing of estrogen and progesterone receptors in breast cancer. J. Clin. Oncol. *28*, 2784–2795.
2. Haffty, B.G., Yang, Q., Reiss, M., Kearney, T., Higgins, S.A., Weidhaas, J., Harris, L., Hait, W., and Toppmeyer, D. (2006). Locoregional relapse and distant metastasis in conservatively managed triple negative early-stage breast cancer. J. Clin. Oncol. *24*, 5652–5657.
3. Lehmann, B.D., Bauer, J.A., Chen, X., Sanders, M.E., Chakravarthy, A.B., Shyr, Y., and Pietenpol, J.A. (2011). Identification of human triple-negative breast cancer subtypes and preclinical models for selection of targeted therapies. J. Clin. Invest. *121*, 2750–2767.
4. Lehmann, B.D., Jovanović, B., Chen, X., Estrada, M.V., Johnson, K.N., Shyr, Y., Moses, H.L., Sanders, M.E., and Pietenpol, J.A. (2016). Refinement of Triple-Negative Breast Cancer Molecular Subtypes: Implications for Neoadjuvant Chemotherapy Selection. PLoS One *11*, e0157368.
5. Sorlie, T., Perou, C.M., Tibshirani, R., Aas, T., Geisler, S., Johnsen, H., Hastie, T., Eisen, M.B., and van de Rijn, M. (2001). Jeffrey S.S.et al. Gene expression patterns of breast carcinomas distinguish tumor subclasses with clinical implications. Proc Natl Acad Sci USA *98*, 10869–10874.
6. Perou, C.M., Sørlie, T., Eisen, M.B., van de Rijn, M., Jeffrey, S.S., Rees, C.A., Pollack, J.R., Ross, D.T., Johnsen, H., Akslen, L.A., et al. (2000). Molecular portraits of human breast tumours. Nature *406*, 747–752.
7. Parker, J.S., Mullins, M., Cheang, M.C.U., Leung, S., Voduc, D., Vickery, T., Davies, S., Fauron, C., He, X., Hu, Z., et al. (2009). Supervised risk predictor of breast cancer based on intrinsic subtypes. J. Clin. Oncol. *27*, 1160–1167.
8. Cancer Genome Atlas Network (2012). Comprehensive molecular portraits of human breast tumours. Nature *490*, 61–70.
9. Curtis, C., Shah, S.P., Chin, S.F., Turashvili, G., Rueda, O.M., Dunning, M.J., Speed, D., Lynch, A.G., Samarajiwa, S., Yuan, Y., et al. (2012). The genomic and transcriptomic architecture of 2,000 breast tumours reveals novel subgroups. Nature *486*, 346–352.
10. Hanahan, D., and Weinberg, R.A. (2011). Hallmarks of cancer: the next generation. Cell *144*, 646–674.
11. Hanahan, D., and Coussens, L.M. (2012). Accessories to the crime: functions of cells recruited to the tumor microenvironment. Cancer Cell *21*, 309–322.
12. Sotiriou, C., and Pusztai, L. (2009). Gene-expression signatures in breast cancer. N. Engl. J. Med. *360*, 790–800.
13. Russnes, H.G., Navin, N., Hicks, J., and Borresen-Dale, A.L. (2011). Insight into the heterogeneity of breast cancer through next-generation sequencing. J. Clin. Invest. *121*, 3810–3818.
14. Stephens, P.J., Tarpey, P.S., Davies, H., Van Loo, P., Greenman, C., Wedge, D.C., Nik-Zainal, S., Martin, S., Varela, I., Bignell, G.R., et al.

(2012). The landscape of cancer genes and mutational processes in breast cancer. Nature *486*, 400–404.

15. Zack, T.I., Schumacher, S.E., Carter, S.L., Cherniack, A.D., Saksena, G., Tabak, B., Lawrence, M.S., Zhsng, C.Z., Wala, J., Mermel, C.H., et al. (2013). Pan-cancer patterns of somatic copy number alteration. Nat. Genet. *45*, 1134–1140.
16. Beroukhim, R., Mermel, C.H., Porter, D., Wei, G., Raychaudhuri, S., Donovan, J., Barretina, J., Boehm, J.S., Dobson, J., Urashima, M., et al. (2010). The landscape of somatic copy-number alteration across human cancers. Nature *463*, 899–905.
17. Gerstung, M., Jolly, C., Leshchiner, I., Dentro, S.C., Gonzalez, S., Rosebrock, D., Mitchell, T.J., Rubanova, Y., Anur, P., Yu, K., et al. (2020). The evolutionary history of 2,658 cancers. Nature *578*, 122–128.
18. Minussi, D.C., Nicholson, M.D., Ye, H., Davis, A., Wang, K., Baker, T., Tarabichi, M., Sei, E., Du, H., Rabbani, M., et al. (2021). Breast tumours maintain a reservoir of subclonal diversity during expansion. Nature *592*, 302–308.
19. Sack, L.M., Davoli, T., Li, M.Z., Li, Y., Xu, Q., Naxerova, K., Wooten, E.C., Bernardi, R.J., Martin, T.D., Chen, T., et al. (2018). Profound tissue specificity in proliferation control underlies cancer drivers and aneuploidy patterns. Cell *173*, 499–514.
20. Davoli, T., Uno, H., Wooten, E.C., and Elledge, S.J. (2017). Tumor aneuploidy correlates with markers of immune evasion and with reduced response to immunotherapy. Science *355*, eaaf8399.
21. Knight, J.F., Sung, V.Y.C., Kuzmin, E., Couzens, A.L., de Verteuil, D.A., Ratcliffe, C.D.H., Coelho, P.P., Johnson, R.M., Samavarchi-Tehrani, P., Gruosso, T., et al. (2018). KIBRA (WWC1) Is a Metastasis Suppressor Gene Affected by Chromosome 5q Loss in Triple-Negative Breast Cancer. Cell Rep. *22*, 3191–3205.
22. Cai, Y., Crowther, J., Pastor, T., Abbasi Asbagh, L., Baietti, M.F., De Troyer, M., Vazquez, I., Talebi, A., Renzi, F., Dehairs, J., et al. (2016). Loss of Chromosome 8p Governs Tumor Progression and Drug Response by Altering Lipid Metabolism. Cancer Cell *29*, 751–766.
23. Liu, Y., Chen, C., Xu, Z., Scuoppo, C., Rillahan, C.D., Gao, J., Spitzer, B., Bosbach, B., Kastenhuber, E.R., Baslan, T., et al. (2016). Deletions linked to TP53 loss drive cancer through p53-independent mechanisms. Nature *531*, 471–475.
24. Taylor, A.M., Shih, J., Ha, G., Gao, G.F., Zhang, X., Berger, A.C., Schumacher, S.E., Wang, C., Hu, H., Liu, J., et al. (2018). Genomic and Functional Approaches to Understanding Cancer Aneuploidy. Cancer Cell *33*, 676–689.e3.
25. Sondka, Z., Bamford, S., Cole, C.G., Ward, S.A., Dunham, I., and Forbes, S.A. (2018). The COSMIC Cancer Gene Census: describing genetic dysfunction across all human cancers. Nat. Rev. Cancer *18*, 696–705.
26. Li, H., Cao, Y., Ma, J., Luo, L., and Ma, B. (2021). Expression and prognosis analysis of GINS subunits in human breast cancer. Medicine (Baltim.) *100*, e24827.
27. Zhang, S., Wu, H., Wang, K., and Liu, M. (2019). STK33/ERK2 signal pathway contribute the tumorigenesis of colorectal cancer HCT15 cells. Biosci. Rep. *39*.
28. Lee, L.J., Papadopoli, D., Jewer, M., Del Rincon, S., Topisirovic, I., Lawrence, M.G., and Postovit, L.M. (2021). Cancer Plasticity: The Role of mRNA Translation. Trends Cancer *7*, 134–145.
29. Savage, P., Blanchet-Cohen, A., Revil, T., Badescu, D., Saleh, S.M.I., Wang, Y., Zuo, D., Liu, L., and Bertos, N.R. (2020). Munoz-Ramos V.,et al. Chemogenomic profiling of breast cancer patient-derived xenografts reveals targetable vulnerabilities for difficult-to-treat tumors. Commun. Biol. *16*, 310.
30. Karlsson, K., Przybilla, M.J., Kotler, E., Khan, A., Xu, H., Karagyozova, K., Sockell, A., Wong, W.H., Liu, K., Mah, A., et al. (2023). Deterministic evolution and stringent selection during preneoplasia. Nature *618*, 383–393.
31. Lips, E.H., Kumar, T., Megalios, A., Visser, L.L., Sheinman, M., Fortunato, A., Shah, V., Hoogstraat, M., Sei, E., Mallo, D., et al. (2022). Genomic analysis defines clonal relationships of ductal carcinoma in situ and recurrent invasive breast cancer. Nat. Genet. *54*, 850–860.
32. Savage, P., Blanchet-Cohen, A., Revil, T., Badescu, D., Saleh, S.M.I., Wang, Y.C., Zuo, D., Liu, L., Bertos, N.R., Munoz-Ramos, V., et al. (2017). A Targetable EGFR-Dependent Tumor-Initiating Program in Breast Cancer. Cell Rep. *21*, 1140–1149.
33. Kuzmin, E., Monlong, J., Martinez, C., Kuasne, H., Kleinman, C.L., Ragoussis, J., Bourque, G., and Park, M. (2021). Inferring Copy Number from Triple-Negative Breast Cancer Patient Derived Xenograft scRNAseq Data Using scCNA. Methods Mol. Biol. *2381*, 285–303.
34. Tirosh, I., Izar, B., Prakadan, S.M., Wadsworth, M.H., 2nd, Treacy, D., Trombetta, J.J., Rotem, A., Rodman, C., Lian, C., Murphy, G., et al. (2016). Dissecting the multicellular ecosystem of metastatic melanoma by single-cell RNA-seq. Science *352*, 189–196.
35. The ORFeome Collaboration (2016). The ORFeome Collaboration: a genome-scale human ORF-clone resource. Nat. Methods *13*, 191–192.
36. Aziz, D., Portman, N., Fernandez, K.J., Lee, C., Alexandrou, S., Llop-Guevara, A., Phan, Z., Yong, A., Wilkinson, A., Sergio, C.M., et al. (2021). Synergistic targeting of BRCA1 mutated breast cancers with PARP and CDK2 inhibition. NPJ Breast Cancer *7*, 111.
37. Shen, L., Pugsley, L., Cencic, R., Wang, H., Robert, F., Naineni, S.K., Sahni, A., Morin, G., Zhang, W., Nijnik, A., et al. (2021). A forward genetic screen identifies modifiers of rocaglate responsiveness. Sci. Rep. *11*, 18516.
38. Alliance of Genome Resources Consortium (2022). Harmonizing model organism data in the Alliance of Genome Resources. Genetics *220*, iyac022.
39. Paysan-Lafosse, T., Blum, M., Chuguransky, S., Grego, T., Pinto, B.L., Salazar, G., Bileschi, M., Bork, P., Bridge, A., Colwell, L., et al. (2023). InterPro in 2022. Nucleic Acids Res. *51*, D418–D427.
40. Jumper, J., Evans, R., Pritzel, A., Green, T., Figurnov, M., Ronneberger, O., Tunyasuvunakool, K., Bates, R., Žídek, A., Potapenko, A., et al. (2021). Highly accurate protein structure prediction with AlphaFold. Nature *596*, 583–589.
41. Oughtred, R., Rust, J., Chang, C., Breitkreutz, B.J., Stark, C., Willems, A., Boucher, L., Leung, G., Kolas, N., Zhang, F., et al. (2021). The BioGRID database: A comprehensive biomedical resource of curated protein, genetic, and chemical interactions. Protein Sci. *30*, 187–200.
42. Luck, K., Kim, D.K., Lambourne, L., Spirohn, K., Begg, B.E., Bian, W., Brignall, R., Cafarelli, T., Campos-Laborie, F.J., Charloteaux, B., et al. (2020). A reference map of the human binary protein interactome. Nature *580*, 402–408.
43. Huttlin, E.L., Bruckner, R.J., Navarrete-Perea, J., Cannon, J.R., Baltier, K., Gebreab, F., Gygi, M.P., Thornock, A., Zarraga, G., Tam, S., et al. (2021). Dual proteome-scale networks reveal cell-specific remodeling of the human interactome. Cell *184*, 3022–3040.e28.
44. Ceccarelli, D.F., Laister, R.C., Mulligan, V.K., Kean, M.J., Goudreault, M., Scott, I.C., Derry, W.B., Chakrabartty, A., Gingras, A.C., and Sicheri, F. (2011). CCM3/PDCD10 heterodimerizes with germinal center kinase III (GCKIII) proteins using a mechanism analogous to CCM3 homodimerization. J. Biol. Chem. *286*, 25056–25064.
45. Branon, T.C., Bosch, J.A., Sanchez, A.D., Udeshi, N.D., Svinkina, T., Carr, S.A., Feldman, J.L., Perrimon, N., and Ting, A.Y. (2018). Efficient proximity labeling in living cells and organisms with TurboID. Nat. Biotechnol. *36*, 880–887.
46. Uhlen, M., Zhang, C., Lee, S., Sjöstedt, E., Fagerberg, L., Bidkhori, G., Benfeitas, R., Arif, M., Liu, Z., Edfors, F., et al. (2017). A pathology atlas of the human cancer transcriptome. Science *357*, eaan2507.
47. Thul, P.J., Åkesson, L., Wiking, M., Mahdessian, D., Geladaki, A., Ait Blal, H., Alm, T., Asplund, A., Björk, L., Breckels, L.M., et al. (2017). A subcellular map of the human proteome. Science *356*, eaal3321.
48. Hart, T., Chandrashekhar, M., Aregger, M., Steinhart, Z., Brown, K.R., MacLeod, G., Mis, M., Zimmermann, M., Fradet-Turcotte, A., Sun, S., et al.

(2015). High-Resolution CRISPR Screens Reveal Fitness Genes and Genotype-Specific Cancer Liabilities. Cell *163*, 1515–1526.

49. Pacini, C., Dempster, J.M., Boyle, I., Gonçalves, E., Najgebauer, H., Karakoc, E., van der Meer, D., Barthorpe, A., Lightfoot, H., Jaaks, P., et al. (2021). Integrated cross-study datasets of genetic dependencies in cancer. Nat. Commun. *12*, 1661.

50. Wang, Y., Yang, W., Pu, Q., Yang, Y., Ye, S., Ma, Q., Ren, J., Cao, Z., Zhong, G., Zhang, X., et al. (2015). The effects and mechanisms of SLC34A2 in tumorigenesis and progression of human non-small cell lung cancer. J. Biomed. Sci. *22*, 52.

51. Lv, Y., Zhang, W., Zhao, J., Sun, B., Qi, Y., Ji, H., Chen, C., Zhang, J., Sheng, J., Wang, T., et al. (2021). SRSF1 inhibits autophagy through regulating Bcl-x splicing and interacting with PIK3C3 in lung cancer. Signal Transduct. Target. Ther. *6*, 108.

52. Horiguchi, H., Xu, H., Duvert, B., Ciuculescu, F., Yao, Q., Sinha, A., McGuinness, M., Harris, C., Brendel, C., Troeger, A., et al. (2022). Deletion of murine Rhoh leads to de-repression of Bcl-6 via decreased KAISO levels and accelerates a malignancy phenotype in a murine model of lymphoma. Small GTPases *13*, 267–281.

53. Bachetti, T., and Ceccherini, I. (2020). Causative and common PHOX2B variants define a broad phenotypic spectrum. Clin. Genet. *97*, 103–113.

54. Mondal, M., Conole, D., Nautiyal, J., and Tate, E.W. (2022). UCHL1 as a novel target in breast cancer: emerging insights from cell and chemical biology. Br. J. Cancer *126*, 24–33.

55. Xue, W., Kitzing, T., Roessler, S., Zuber, J., Krasnitz, A., Schultz, N., Revill, K., Weissmueller, S., Rappaport, A.R., Simon, J., et al. (2012). A cluster of cooperating tumor-suppressor gene candidates in chromosomal deletions. Proc. Natl. Acad. Sci. USA *109*, 8212–8217.

56. Wistuba, I.I., Behrens, C., Virmani, A.K., Mele, G., Milchgrub, S., Girard, L., Fondon, J.W., 3rd, Garner, H.R., McKay, B., Latif, F., et al. (2000). High resolution chromosome 3p allelotyping of human lung cancer and preneoplastic/preinvasive bronchial epithelium reveals multiple, discontinuous sites of 3p allele loss and three regions of frequent breakpoints. Cancer Res. *60*, 1949–1960.

57. Weigman, V.J., Chao, H.H., Shabalin, A.A., He, X., Parker, J.S., Nordgard, S.H., Grushko, T., Huo, D., Nwachukwu, C., Nobel, A., et al. (2012). Basal-like Breast cancer DNA copy number losses identify genes involved in genomic instability, response to therapy, and patient survival. Breast Cancer Res. Treat. *133*, 865–880.

58. International Stem Cell Initiative; Amps, K., Andrews, P.W., Anyfantis, G., Armstrong, L., Avery, S., Baharvand, H., Baker, J., Baker, D., Munoz, M.B., et al. (2011). Screening ethnically diverse human embryonic stem cells identifies a chromosome 20 minimal amplicon conferring growth advantage. Nat. Biotechnol. *29*, 1132–1144.

59. Gruosso, T., Gigoux, M., Manem, V.S.K., Bertos, N., Zuo, D., Perlitch, I., Saleh, S.M.I., Zhao, H., Souleimanova, M., Johnson, R.M., et al. (2019). Spatially distinct tumor immune microenvironments stratify triple-negative breast cancers. J. Clin. Invest. *129*, 1785–1800.

60. Davoli, T., Xu, A.W., Mengwasser, K.E., Sack, L.M., Yoon, J.C., Park, P.J., and Elledge, S.J. (2013). Cumulative haploinsufficiency and triplosensitivity drive aneuploidy patterns and shape the cancer genome. Cell *155*, 948–962.

61. Sheltzer, J.M., Ko, J.H., Replogle, J.M., Habibe Burgos, N.C., Chung, E.S., Meehl, C.M., Sayles, N.M., Passerini, V., Storchova, Z., and Amon, A. (2017). Single-chromosome Gains Commonly Function as Tumor Suppressors. Cancer Cell *31*, 240–255.

62. Ben-David, U., and Amon, A. (2020). Context is everything: aneuploidy in cancer. Nat. Rev. Genet. *21*, 44–62.

63. Anders, K.R., Kudrna, J.R., Keller, K.E., Kinghorn, B., Miller, E.M., Pauw, D., Peck, A.T., Shellooe, C.E., and Strong, I.J.T. (2009). A strategy for constructing aneuploid yeast strains by transient nondisjunction of a target chromosome. BMC Genet. *10*, 36.

64. Ravichandran, M.C., Fink, S., Clarke, M.N., Hofer, F.C., and Campbell, C.S. (2018). Genetic interactions between specific chromosome copy number alterations dictate complex aneuploidy patterns. Genes Dev. *32*, 1485–1498.

65. Goudreault, M., D'Ambrosio, L.M., Kean, M.J., Mullin, M.J., Larsen, B.G., Sanchez, A., Chaudhry, S., Chen, G.I., Sicheri, F., Nesvizhskii, A.I., et al. (2009). A PP2A phosphatase high density interaction network identifies a novel striatin-interacting phosphatase and kinase complex linked to the cerebral cavernous malformation 3 (CCM3) protein. Mol. Cell. Proteomics *8*, 157–171.

66. Kean, M.J., Ceccarelli, D.F., Goudreault, M., Sanches, M., Tate, S., Larsen, B., Gibson, L.C.D., Derry, W.B., Scott, I.C., Pelletier, L., et al. (2011). Structure-function analysis of core STRIPAK Proteins: a signaling complex implicated in Golgi polarization. J. Biol. Chem. *286*, 25065–25075.

67. Valentino, M., Dejana, E., and Malinverno, M. (2021). The multifaceted PDCD10/CCM3 gene. Genes Dis. *8*, 798–813.

68. Zhang, Y., Tang, W., Zhang, H., Niu, X., Xu, Y., Zhang, J., Gao, K., Pan, W., Boggon, T.J., Toomre, D., et al. (2013). A network of interactions enables CCM3 and STK24 to coordinate UNC13D-driven vesicle exocytosis in neutrophils. Dev. Cell *27*, 215–226.

69. Jenny Zhou, H., Qin, L., Zhang, H., Tang, W., Ji, W., He, Y., Liang, X., Wang, Z., Yuan, Q., Vortmeyer, A., et al. (2016). Endothelial exocytosis of angiopoietin-2 resulting from CCM3 deficiency contributes to cerebral cavernous malformation. Nat. Med. *22*, 1033–1042.

70. Fujii, M., Yan, J., Rolland, W.B., Soejima, Y., Caner, B., and Zhang, J.H. (2013). Early brain injury, an evolving frontier in subarachnoid hemorrhage research. Transl. Stroke Res. *4*, 432–446.

71. Coelho, P.P., Hesketh, G.G., Pedersen, A., Kuzmin, E., Fortier, A.M.N., Bell, E.S., Ratcliffe, C.D.H., Gingras, A.C., and Park, M. (2022). Endosomal LC3C-pathway selectively targets plasma membrane cargo for autophagic degradation. Nat. Commun. *13*, 3812.

72. Liu, G., Knight, J.D.R., Zhang, J.P., Tsou, C.C., Wang, J., Lambert, J.P., Larsen, B., Tyers, M., Raught, B., Bandeira, N., et al. (2016). Data Independent Acquisition analysis in ProHits 4.0. J. Proteomics *149*, 64–68.

73. Talevich, E., Shain, A.H., Botton, T., and Bastian, B.C. (2016). CNVkit: Genome-Wide Copy Number Detection and Visualization from Targeted DNA Sequencing. PLoS Comput. Biol. *12*, e1004873.

74. Hart, T., Tong, A.H.Y., Chan, K., Van Leeuwen, J., Seetharaman, A., Aregger, M., Chandrashekhar, M., Hustedt, N., Seth, S., Noonan, A., et al. (2017). Evaluation and Design of Genome-Wide CRISPR/SpCas9 Knockout Screens. G3 (Bethesda) *7*, 2719–2727.

75. Leshchiner, I., Mroz, E.A., Cha, J., Rosebrock, D., Spiro, O., Bonilla-Velez, J., Faquin, W.C., Lefranc-Torres, A., Lin, D.T., Michaud, W.A., et al. (2023). Inferring early genetic progression in cancers with unobtainable premalignant disease. Nat. Cancer *4*, 550–563.

76. Liu, X., Krawczyk, E., Suprynowicz, F.A., Palechor-Ceron, N., Yuan, H., Dakic, A., Simic, V., Zheng, Y.L., Sripadhan, P., Chen, C., et al. (2017). Conditional reprogramming and long-term expansion of normal and tumor cells from human biospecimens. Nat. Protoc. *12*, 439–451.

77. Robinson, M.D., and Oshlack, A. (2010). A scaling normalization method for differential expression analysis of RNA-seq data. Genome Biol. *11*, R25.

78. Ritchie, M.E., Phipson, B., Wu, D., Hu, Y., Law, C.W., Shi, W., and Smyth, G.K. (2015). limma powers differential expression analyses for RNA-sequencing and microarray studies. Nucleic Acids Res. *43*, e47.

79. Perkins, D.N., Pappin, D.J., Creasy, D.M., and Cottrell, J.S. (1999). Probability-based protein identification by searching sequence databases using mass spectrometry data. Electrophoresis *20*, 3551–3567.

80. Eng, J.K., Jahan, T.A., and Hoopmann, M.R. (2013). Comet: an open-source MS/MS sequence database search tool. Proteomics *13*, 22–24.

81. Shteynberg, D., Deutsch, E.W., Lam, H., Eng, J.K., Sun, Z., Tasman, N., Mendoza, L., Moritz, R.L., Aebersold, R., and Nesvizhskii, A.I. (2011).

iProphet: multi-level integrative analysis of shotgun proteomic data improves peptide and protein identification rates and error estimates. Mol. Cell. Proteomics *10*, M111.007690.

82. Teo, G., Liu, G., Zhang, J., Nesvizhskii, A.I., Gingras, A.C., and Choi, H. (2014). SAINTexpress: improvements and additional features in Significance Analysis of INTeractome software. J. Proteomics *100*, 37–43.

83. Bloh, K., Kanchana, R., Bialk, P., Banas, K., Zhang, Z., Yoo, B.C., and Kmiec, E.B. (2021). Deconvolution of Complex DNA Repair (DECODR): Establishing a Novel Deconvolution Algorithm for Comprehensive Analysis of CRISPR-Edited Sanger Sequencing Data. CRISPR J. *4*, 120–131.

84. O'Flanagan, C.H., Campbell, K.R., Zhang, A.W., Kabeer, F., Lim, J.L.P., Biele, J., Eirew, P., Lai, D., McPherson, A., Kong, E., et al. (2019). Dissociation of solid tumor tissues with cold active protease for single-cell RNA-seq minimizes conserved collagenase-associated stress responses. Genome Biol. *20*, 210.

85. Nik-Zainal, S., Van Loo, P., Wedge, D.C., Alexandrov, L.B., Greenman, C.D., Lau, K.W., Raine, K., Jones, D., Marshall, J., Ramakrishna, M., et al. (2012). The life history of 21 breast cancers. Cell *149*, 994–1007.

86. ICGC/TCGA Pan-Cancer Analysis of Whole Genomes Consortium (2020). Pan-cancer analysis of whole genomes. Nature *578*, 82–93.

87. Kaufmann, T.L., Petkovic, M., Watkins, T.B.K., Colliver, E.C., Laskina, S., Thapa, N., Minussi, D.C., Navin, N., Swanton, C., Van Loo, P., et al. (2022). MEDICC2: whole-genome doubling aware copy-number phylogenies for cancer evolution. Genome Biol. *23*, 241.

88. Dentro, S.C., Wedge, D.C., and Van Loo, P. (2017). Principles of Reconstructing the Subclonal Architecture of Cancers. Cold Spring Harb. Perspect. Med. *7*, a026625.

89. Wang, R., Lin, D.Y., and Jiang, Y. (2020). A Normalization and Copy-Number Estimation Method for Single-Cell DNA Sequencing. Cell Syst. *10*, 445–452.e6.

90. Bourgey, M., Dali, R., Eveleigh, R., Chen, K.C., Letourneau, L., Fillon, J., Michaud, M., Caron, M., Sandoval, J., Lefebvre, F., et al. (2019). GenPipes: an open-source framework for distributed and scalable genomic analyses. GigaScience *8*, giz037.

91. Zaccaria, S., and Raphael, B.J. (2021). Characterizing allele- and haplotype-specific copy numbers in single cells with CHISEL. Nat. Biotechnol. *39*, 207–214.

STAR★METHODS

KEY RESOURCES TABLE

REAGENT or RESOURCE	SOURCE	IDENTIFIER
Antibodies		
V5 anti-mouse	Abcam	Cat#27671; RRID:AB_471093
GAPDH anti-rabbit	Santa Cruz Biotechnology	Cat#sc-25778; RRID:AB_10167668
FLAG anti-mouse	Sigma	Cat#F3165; RRID:AB_259529
C4orf19 anti-rabbit	GeneTex	Cat#GTX106538; RRID:AB_1949787
Anti-mouse-680	Mandel Scientific	Cat#LIC-926-68070
Anti-rabbit-800	Mandel Scientific	Cat#LIC-926-32211
Ki67 anti-rabbit	Roche	Cat#790–4286; RRID:AB_2631262
Pan-Keratin anti-mouse	Roche	Cat# 760–2595; RRID:AB_2941938
FITC anti-human EpCAM (clone VU-1D9)	Thermo Fisher Scientific	Cat#A15755
PE/Cy7 anti-mouse H2Kd (clone SF1-1.1)	Biolegend	Cat#116622
IgG, HRP-linked anti-mouse	NEB	Cat#7076S
IgG, HRP-linked anti-rabbit	NEB	Cat#7074S
Bacterial and virus strains		
MISSION® TRC3 Human ORF collection	McGill Platform for Cell Perturbation	N/A
Biological samples		
GCRC1735 PDX	Goodman Cancer Institute	N/A
Mammoplasty reduction samples	McGill University Health Center	N/A
3T3-J2 Irradiated Feeder Cells	STEMCELL Technologies	Cat#100-0353
Critical commercial assays		
RNAscope 2.5 HD Assay	ACD Bio	Cat#322360
RNAscope probe Hs-RBPJ	ACD Bio	Cat#448661
Tumor Dissociation Kit	Miltenyi	Cat#130-095-929
Mouse cell depletion kit	Miltenyi	Cat# 130-104-694
Feeder Removal MicroBeads, mouse	Miltenyi	Cat#130-095-531
Lonza Mycoalert Mycoplasma Detection Kit	Lonza	Cat#LT07-318
EZ-PCR Mycoplasma Detection Kit	Sartorius	Cat#20-700-20
Roche High Pure PCR Template Preparation Kit	Roche	Cat#11796828001
Roche PCR Product Purification Kit	Roche	Cat#11732668001
Chromium Next GEM Single Cell 3′ GEM, Library & Gel Bead Kit v3.1	10x Genomics	Cat#PN-1000121
Chromium Next GEM Chip G Single Cell Kit	10x Genomics	Cat#PN-1000120
Beckman Coulter SPRIselect	Fisher scientific	Cat#B23317
KAPA Biosystems Library Quantification Kit for Illumina platforms	KAPA Biosystems	Cat#KR0405
Chromium Single Cell DNA Reagent Kit	10x Genomics	N/A
Deposited data		
Single cell DNA- and RNA-sequencing data	This paper	ENA: PRJEB62760
BioID data	This paper	MassIVE: MSV000090947
BioID data	This paper	ProteomeXchange: PXD039042
Experimental models: Cell lines		
MCF10A	ATCC	Cat#CRL-10317
MDA-MB-468	ATCC	Cat#HTB-132
HEK293T	ATCC	Cat#CRL-3216

(*Continued on next page*)

**Continued**		
REAGENT or RESOURCE	SOURCE	IDENTIFIER
GCRC1735 PDX-derived cell line	Goodman Cancer Institute	N/A
GCRC1915 PDX-derived cell line	Goodman Cancer Institute	N/A
Oligonucleotides		
All oligonucleotides are listed in the STAR Methods section	This paper	N/A
Software and algorithms		
MATLAB	Mathworks	https://www.mathworks.com/products/matlab.html
scSNA	Kuzmin et al.[33]	https://github.com/jmonlong/scCNAutils
Treeview 3.0	Zenodo	https://doi.org/10.5281/zenodo.160573
Gistic2.0	GenePattern	https://www.genepattern.org/#gsc.tab=0
limma R package	Ritchie et al.[71]	https://doi.org/10.1093/nar/gkv007
CNVkit	Talevich et al.[72]	https://doi.org/10.1371/journal.pcbi.1004873
edgeR's TMM algorithm	Robinson et al.[73]	https://doi.org/10.1186/gb-2010-11-3-r25
fgsea	Bioconductor	http://bioconductor.org/packages/fgsea/
Trans-Proteomic Pipeline (v4.7 POLAR VORTEX rev 1) and iProphet pipeline	Shteynberg et al.[74]	https://doi.org/10.1074/mcp.M111.007690
g:Profiler	g:Profiler	https://biit.cs.ut.ee/gprofiler/gost
Fiji (v.2.3)	NIH	https://imagej.net/software/fiji/downloads
cBioPortal	Memorial Sloan Kettering Cancer Center	https://www.cbioportal.org/
Cell Ranger Pipeline version 3.0.1	10XGenomics	https://www.10xgenomics.com/support/software/cell-ranger/latest
R package Seurat (v3.2.3)	Satija lab	https://satijalab.org/seurat/articles/install.html
Battenberg (v2.2.9)	Wedge lab	https://github.com/Wedge-lab/battenberg
Beagle 5.1	Brian Browning	https://faculty.washington.edu/browning/beagle/b5_1.html
MEDICC2 (v0.3)	Kaufmann et al.[75]	https://doi.org/10.1186/s13059-022-02794-9
DPClust (v2.2.8)	Wedge lab	https://github.com/Wedge-lab/dpclust
emcee sampler	Arxiv	https://arxiv.org/abs/1202.3665
MutationTimeR	Gerstung lab	https://github.com/gerstung-lab/MutationTimeR
Samtools	Samtools	https://www.htslib.org/
Mutect2	BroadInstitute	https://gatk.broadinstitute.org/hc/en-us/articles/360037593851-Mutect2
BVAtools basefreq		https://bitbucket.org/mugqic/bvatools/src/master/
CHISEL	Raphael group	https://github.com/raphael-group/chisel

RESOURCE AVAILABILITY

Lead contact

Further information and requests for resources and reagents should be directed to and will be fulfilled by the lead contact, Elena Kuzmin (elena.kuzmin@concordia.ca).

Materials availability

PDX-derived cell lines, mutant cell lines and plasmids generated in this study are available upon request.

Data and code availability

- Single cell DNA- and RNA-sequencing data have been deposited in the European Nucleotide Archive (ENA): PRJEB62760 (https://www.ebi.ac.uk/ena/browser/view/PRJEB62760). Data have been deposited as a complete submission to the MassIVE and assigned the accession number MSV000090947: https://massive.ucsd.edu/ProteoSAFe/static/massive.jsp. The ProteomeXchange accession is PXD039042. All supplementary tables are available at Mendeley Data, V1, https://doi.org/10.17632/gs2cchr2yf.1. Any remaining data pertaining to this manuscript is available from the corresponding author upon request.
- This paper does not report original code.
- Any additional information required to reanalyze the data reported in this work paper is available from the lead contact upon request.

EXPERIMENTAL MODEL AND STUDY PARTICIPANT DETAILS

Isolation of normal breast epithelial single Cell suspension

All tissue was collected with informed consent under REB-approved protocols at the McGill University Health Center. Two patients age of 46 and 18 years old undergoing bilateral mammoplasty reduction due to hypertrophy of the breast with diagnosis and/or management at McGill University Health Center, Montreal, QC, Canada were recruited for this study. 2,000–3,000 mm^3 surgically removed breast epithelial tissue was harvested and kept on ice in transport medium: RPMI 1640, 50 μg/mL gentamycin, 100 U/ml Pen/Strep, 2.5 μg/mL Fungizone until sample processing. The tissue dissociation was achieved as previously described.[33] Briefly, the tissue fragment was minced in ~1 mL cold DMEM and MidiMACS Starting Kit (LS) was used as per manufacturing instructions. Minced tissue was collected in a sterile gentleMACS C tube and enzyme A, enzyme R and enzyme H were added from the Tumor Dissociation Kit. GentleMACS Octo Dissociator with Heaters, program was run (1 h, mild speed, 37°C) to begin the mechanical and enzymatic digestion process. The mix was incubated on ice for 3 min to allow for gravity sedimentation and the oily layer was aspirated. 3 mL of cell suspension was run through 70 μm strainer and collected. The remaining undigested tissue fragments were loaded into the gentleMACS Octo Dissociator, program (1 min, high speed, room temperature) to continue the mechanical and enzymatic digestion process. The cell suspension was passed through 70 μm strainer and collected. The strainer was washed with 10 mL PBS and combined with the filtrate, centrifuge for 10 min, 1,500 rpm. The cell pellet was resuspended in 500 μL Complete DMEM and Trypan Blue staining was used to quantify cell number and viability. Red blood cells were removed by aspirating the supernatant and adding 3 mL ACK lysing buffer and incubating at room temperature for 5 min. Then, 7 mL PBS were added and the suspension was centrifuged at 1,200 rpm for 4 min. The cell pellet was then resuspended in 500 μL complete DMEM. Single cell RNA sequencing was conducted if the cell viability exceeded 60%.

Cell culture

MCF10A cell line was obtained from the ATCC and cultured in DMEM/Ham's F12 medium, 20 ng/mL hEGF, 100 ng/mL cholera toxin, 10 μg/mL bovine insulin, 500 ng/mL hydrocortisone, 5% horse serum (HS), 50 μg/mL gentamicin. HEK293T cell line was cultured in DMEM with 10% fetal bovine serum (FBS).

Patient-derived xenograft derived cell lines (GCRC1735, GCRC1915) were isolated from the respective PDXs. PDX tumor fragments were minced and digested as previously described.[33] Cancer epithelial cells were established using a Conditional reprogramming protocol as previously described.[76] Briefly, after tumor fragments digestion using MidiMACS Starting Kit and Tissue Dissociation Kit, single-human epithelial cancer cells were transferred to a dish containing lethally irradiated 3T3-J2 cells (1 × 106 cells) and cultured with F- media (DMEM (Gibco) and F-12 Nutrient Mixture (Ham) (Gibco-) (1:4), 5% FBS (Life Technologies), 0.4 μg/mL Hydrocortisone (Sigma-Aldrich), 5 μg/mL Insulin (Gibco-), 8.4 ng/mL Cholera toxin (Sigma-Aldrich), 10 ng/mL Epidermal growth factor (BPS bioscience), 10 μmol/L Y-27632 (Abmole), 50 μg/mL Gentamicin (Gibco), 1% P/S (Thermo Fisher Scientific), Amphotericin B (1 μg/mL) (Thermo Fisher Scientific). After five passages of coculture, murine irradiated 3T3-J2 cells were removed using a Feeder Removal MicroBeads kit (Miltenyi), and epithelial cancer cells were expended in F-media. PDX-derived cell lines were cultured in F media: 5% fetal bovine serum, 400 ng/mL hydrocortisone, 5 μg/mL insulin, 8.4 ng/mL cholera toxin, 10 ng/mL hEGF, 10 μM Y-27632 (ROCK inhibitor), 50 μg/mL gentamicin.

All cell lines used were routinely tested for Mycoplasma (Lonza Mycoalert and EZ-PCR Mycoplasma Detection Kit) and were authenticated using short tandem repeat analysis. The human origin of PDX-derived cell lines was validated by flow cytometry using FITC anti-human EpCAM antibody clone VU-1D9 and (Thermo Fisher Scientific, #A15755) and PE/Cy7 anti-mouse H2Kd antibody clone SF1-1.1 (Biolegend, # 116622). All cells were maintained at 37°C, 5% CO_2.

METHOD DETAILS

Single and dual-gene overexpression assay

For generation of stable *c4orf19*, *RBPJ*, *SEPSECS*, *SEL1L3*, *KCNIP4*, *TBC1D19*, *RELL1*, *SLC34A2*, *STIM2*, *LGI2*, *PI4K2B* and *CCKAR* overexpression cells, lentiviral ORF vectors were retrieved from the arrayed MGC premier human lentiviral ORF (Sigma) (ccsb ID, blasticidin resistant) and MISSION TRC3 Human ORF collection (Sigma) (TRCN ID, puromycin resistant) obtained from

the McGill Platform for Cell Perturbation (MPCP). The following lentiviral ORF vectors were used: *c4orf19* (ccsbBroad304_03572, TRCN0000469204), *RBPJ* (ccsbBroad304_06435, TRCN0000470066), *SEPSECS* (ccsbBroad304_11945), *SEL1L3* (ccsbBroad304_11701, TRCN0000479888), *KCNIP4* (ccsbBroad304_09030, TRCN0000474912), *TBC1D19* (TRCN0000468467), *RELL1* (TRCN0000476648), *SLC34A2* (TRCN0000476745), *STIM2* (TRCN0000477969), *LGI2* (TRCN0000481617), *PI4K2B* (TRCN0000489163), *CCKAR* (TRCN0000489014, TRCN0000491970), *CDKN1A* (ccsbBroad304_00282, TRCN0000471863), *CDKN1B* (ccsbBroad304_05980, TRCN0000475049) and GFP control vector pLX317-GFP and pLX304-GFP. All overexpression constructs were verified by a combination of restriction digest with *XhoI* (Thermo FD0694) and Sanger sequencing. Sequencing primer: (5′-3′) CTTGGTTC ATTCTCAAGCCTC. *XhoI* digest was conducted by an incubation of 100 ng plasmid DNA with 0.5 μl *XhoI* enzyme for 10 min, 37°C followed by 10 min, 80°C and resolved on 0.8% agarose gel. Viral particles were produced by co-expressing ORF or control constructs with packaging plasmids psPAX2 and pMD2.G in HEK-293T cells using lipofectamine 2000 transfection protocol. Media containing viral particles were collected and passed through a 0.45 μm filter. Cells were treated with virus in media containing 8 μg/mL polybrene. Twenty-four hours after transduction cells were recovered for another 24 h and then MCF10A were selected in 3 μg/mL puromycin dihydrochloride (Sigma) for 48 h or 10 μg/mL blasticidin (Gibco) for 72 h for; GCRC1735 in 5 μg/mL puromycin dihydrochloride for 48 h or 7.5 μg/mL blasticidin for 72 h and GCRC1915 in 5 μg/mL puromycin dihydrochloride for 48 h or 10 μg/mL blasticidin for 72 h. MOI was determined for each construct for each cell line and ~0.3 MOI was used for all constructs ensuring one integrant per cell. Viral transductions with the respective vectors were carried out sequentially. All single gene overexpression mutant cells were constructed such that they overexpressed each ORF under one of the selection markers and GFP was then under the second selection marker. All double gene overexpression mutant cells were constructed such that they overexpressed each ORF under one of the selection markers and the second ORF was then under the second selection marker. Confluence was measured by IncuCyte. Briefly, 4000 MCF10A and GCRC1915 cells and 2000 GCRC1735 cells were plated per well representing 20% confluence at the start of the experiment. Imaging was done at 4 h intervals for a duration of 5–6 days. Each mutant cell line was plated in three wells per plate for a total of three technical replicates. The experiment was repeated for a total of three independent biological replicates. *CDKN1A* and *CDKN1B* were included as positive controls, which are known to lead to a severe proliferation defect when overexpressed.[19]

Genome-wide pooled overexpression screen

The pooled MISSION TRC3 LentiORF collection (Sigma) provided by the McGill Platform for Cell Perturbation (MPCP) was used to infect MCF10A (10^8) and GCRC1735 (1.5×10^8) cell lines. Cells were treated with virus in media containing 8 μg/mL polybrene for 24 h. Viral supernatant was removed and the media was refreshed recovering the cells for 48 h and then MCF10A and GCRC1735 were selected in 3 or 5 μg/mL puromycin dihydrochloride for 48 h, respectively. MOI ~0.3 MOI was used for the screens and 1000x coverage was maintained at each step of the screen for both cell lines. Following 6–8 doublings, genomic DNA was isolated using the Roche High Pure PCR Template Preparation Kit followed by an RNase A treatment. One microgram of DNA was then used in 48 2-step PCR reactions with barcoded Illumina sequencing primers and then with P5/P7 primers. The reactions were then purified using the Roche PCR Purification Kit. Samples were then sequenced at The Center for Applied Genomics at Toronto Sick-Kids hospital on the Illumina HiSeq 2500 platform. The 50-base kit with 62 cycles and single-end reads was used to obtain the exact read-length needed for the library vector. Sequences were then deconvoluted. For all downstream analyses, we only included genes with a read count higher than 100 in T0 samples (MCF10A_T0 and GCRC1735_T0). Raw counts were normalized using edgeR's TMM algorithm[77] and were then transformed to $\log_2$-counts per million (logCPM) using the voom function implemented in the limma R package.[78] To assess differences in gene expression levels, we fitted a linear model using limma's lmfit function. Nominal p values were corrected for multiple testing using the Benjamini-Hochberg method. Genomic heatmaps of $\log_2$ fold-changes were created using CNVkit.[73]

TCGA data computational analysis

Patient copy number data were obtained from a TCGA Breast Invasive Carcinoma (BRCA) (n = 2199) using Firehose Broad GDAC (https://gdac.broadinstitute.org/; accessed on 31 July 2016). Frequencies of gene deletions were derived from the single nucleotide polymorphism array dataset (genome_wide_snp_6-segmented_scna_minus_germline_cnv_hg19) and analyzed by GISTIC2.0 (Mermel et al., 2011). Parameters used for analysis were: reference genome build hg19; amplification threshold 0.1; deletion threshold −0.1; join segment size 4; qv threshold 0.25; remove X chromosome yes; cap value 1.5; confidence level 95; broad analysis yes; broad length cut-off 0.5; maximum samples per segments per sample 2000; arm peel-off yes. PAM50 annotation was obtained from a previous study.[8] *TP53* mutations were obtained from cBioPortal and missense mutations were annotated by IARC filename: functionalAssessmentIARC TP53 Database, R18.xlsx. LOF mutations were considered: Frame_Shift_Del, Frame_Shift_Ins, Nonsense_Mutation, Splice_Site and Missense_Mutation if there were more cases of LOF than GOF.

Patient copy number data were obtained from a TCGA Lung Squamous Cell Carcinoma (LUSC, n = 1032), Testicular Germ Cell Tumors (TGCT, n = 304), Ovarian Serous Carcinoma (OV, n = 1168), Esophageal carcinoma (ESCA, n = 373), Cervical Squamous Cell Carcinoma and Endocervical Adenocarcinoma (CESC, n = 586), Mesothelioma (MESO, n = 172), Rectum adenocarcinoma (READ, n = 316), Stomach Adenocarcinoma (STAD, n = 904), Breast Invasive Carcinoma (BRCA, n = 2199), Colon Adenocarcinoma and Rectum Adenocarcinoma (COADREAD, n = 1234), Colon Adenocarcinoma (COAD, n = 918), Head and Neck Squamous Cell Carcinoma (HNSC, n = 1089) and Uterine Corpus Endometrial Carcinoma (UCEC, n = 1089) (https://gdac.broadinstitute.org/;

accessed on 31 July 2016). Frequencies of gene deletions were derived from the single nucleotide polymorphism array dataset (genome_wide_snp_6-segmented_scna_minus_germline_cnv_hg19) and analyzed by GISTIC2.0 (Mermel et al., 2011). Parameters used for analysis were: reference genome build hg19; amplification threshold 0.1; deletion threshold −0.1; join segment size 4; qv threshold 0.25; remove X chromosome yes; cap value 1.5; confidence level 95; broad analysis yes; broad length cut-off 0.5; maximum samples per segments per sample 2000; arm peel-off yes. Higher amplification and deletion thresholds than above were used to increase stringency for the pan-cancer analysis.

TCGA gene expression dataset from breast cancer invasive ductal carcinoma was used for differential gene expression (Figures 1G and 1H). For all downstream analyses, excluded lowly expressed genes with an average read count lower than 10 were excluded from all samples. Raw counts were normalized using edgeR's TMM algorithm and were then transformed to log_2-counts per million (logCPM) using the voom function implemented in the limma R package. To assess differences in gene expression levels, we fitted a linear model using limma's lmfit function. To assess the effect of aneuploidy on the differences in gene expression levels, we used the aneuploidy score as covariate. Nominal p values were corrected for multiple testing using the Benjamini-Hochberg method. Gene Set enrichment analysis based on pre-ranked gene list was performed using the R package fgsea (http://bioconductor.org/packages/fgsea/). Default parameters were used.

Immunoprecipitation and western blot

MCF10A cells were plate at a density of 1.5 × 10^6/10cm-dish and transfected with 4 μg of candidate interactors using Lipofectamine (ThermoFisher, 18324012) and Plus (ThermoFisher, 11514015) transfection reagents. For this DNA constructs were incubated with 8μL Plus reagent in 500μL Opti-MEM (ThermoFisher,11058-021), Lipofectamine reagent was incubated separately in another 500μL Opti-MEM, for 15min; following initial incubation, the solutions were mixed and incubated together for another 15min. The transfection mixture was added dropwise to pre-washed cells containing 2mL Opti-MEM, and incubated for another 3h at 37°C, at which point the transfection solution was removed, and cells were returned to normal growth media. Following 24hrs, cells were harvested in lysis buffer (50 mM HEPES, 150 mM NaCl, 1.5 mM $MgCl_2$, 1 mM EGTA, 1% Triton X-100, 10% glycerol, 1 mM PMSF, 1 mM Na_3VO_4, 1 mM NaF, 10 μg/mL aprotinin and 10 μg/mL leupeptin, pH 7.4). Lysates were pre-cleared with 30 μL of either protein-A-sepharose (GE Healthcare, 17-5280-01) or protein-G-sepharose beads (GE Healthcare, 17-0618-01) for 1h at 4°C. 1500 μg of protein was then incubated with either 1.8 μL (~5 μg) Anti-V5 primary antibody (Abcam, ab27671), 1.25 μL (~5 μg) Anti-FLAG primary antibody (Sigma, F3165) or 1.5μL (~5 μg) mouse-IgG negative control and either 40 μL of protein-A or protein-G-sepharose beads overnight at 4°C. Beads with bound proteins were washed three times in lysis buffer plus inhibitors, and eluted by boiling in SDS sample buffer. Eluted proteins and 50 μg of protein from whole cell lysate were resolved in 4–15% NuPAGE gradient gel (ThermoFisher, NP0335) using MOPS running buffer (ThermoFisher, NP000102). Proteins were transferred on PVDF Odyssey membranes (MilliporeSigma) using a Mini Trans-Blot System from Bio-Rad. Detection and quantification of protein levels was performed on the Odyssey IR imaging System (Li-COR Biosciences) using fluorescently labeled secondary antibodies, anti-mouse-680 (Mandel Scientific, LIC-926-68070) or anti-rabbit-800 (Mandel Scientific, LIC-926-32211).

miniTurboID

Gateway cloning was used to clone C4orf19 (ccsbBroadEn_03572) from pDONR223 to pSTV6-miniTurbo. MCF10A cells were transduced with lentivirus backbone containing pSTV6-C4orf19-3xFLAG-miniTurbo or pSTV6-GFP-3xFLAG-miniTurbo and selected in media containing puromycin. Cells were grown to ~70% confluency and bait expression and biotin labeling was induced simultaneously (0.5 μg/mL doxycycline, 40 μM biotin). Western blot was conducted to ensure C4orf19-3xFLAG-miniTurbo expression and biotinylation. Cells were harvested in lysis buffer (50 mM Tris-HCl, pH 8.0, 150 mM NaCl, 1% Nonidet P-40 substitute (NP40; IGEPAL-630), 0.1% SDS, 0.5% sodium deoxycholate, 1 mM PMSF, 1 mM Na_3VO_4, 1 mM NaF, 10 μg/mL aprotinin and 10 μg/mL leupeptin, pH 7.4). Lysates were boiled in SDS sample buffer and 30 μg of protein from whole cell lysate were resolved in 4–15% NuPAGE gradient gel (ThermoFisher, NP0335) using MOPS running buffer (ThermoFisher, NP000102). Proteins were transferred on PVDF Odyssey membranes (MilliporeSigma) using a Mini Trans-Blot System from Bio-Rad. Anti-Flag (1:1000, Sigma, F3165) and Anti-GAPDH (1:1000, Santa Cruz sc-25778) primary antibodies were used. Anti-Rabbit (NEB #7076S) and anti-mouse (NEB #7074S) secondary antibodies were used 1:10,000 in blocking solution. Streptavidin-HRP conjugate (Millipore Sigma #RPN1231VS, 1:5000) was used and visualized using Immobilon Forte Western HRP substrate (Millipore Sigma #WBLUF0500) using the ChemiDoc.

When the desired time point was chosen, cells were grown to ~70% confluency and bait expression and biotin labeling was induced simultaneously (0.5 μg/mL doxycycline, 40 μM biotin). After 4 h, cells were rinsed and scraped into 1 mL of PBS. Cells were collected by centrifugation (500 × g for 3 min) and stored at −80°C until further processing.

Cell pellets were thawed on ice and resuspended in lysis buffer containing 50 mM Tris-HCl, pH 7.5, 150 mM NaCl, 1% Nonidet P-40 substitute (NP40; IGEPAL-630), 0.4% SDS, 1 mM $MgCl_2$, 1 mM EGTA, 0.5 mM EDTA, 0.4% sodium deoxycholate, benzonase & protease inhibitors at a ratio of 10:1 (w/v). Cells were lysed with 15 s of sonication (5 s on, 3 s off) at 30% amplitude on a Q500 Sonicator with an 1/8″ Microtip and were rotated end-over-end at 4°C for 20 min. Cell debris was pelleted via centrifugation at 15,000 × g for 15 min at 4°C. Supernatants were incubated with 25 μL (packed bead volume) of streptavidin-Sepharose beads (GE) with rotation for 3 h at 4°C. Beads were pelleted at 500 × g for 2 min, transferred to new tubes and resuspended in 500 μL of fresh lysis buffer.

Beads were washed once with SDS wash buffer (50 mM Tris-HCl, pH 7.5, 2% SDS), twice with lysis buffer, once with TNNE wash buffer (50 mM Tris-HCl, pH 7.5, 150 mM NaCl, 1 mM EDTA, 0.1% NP-40), and thrice with 50 mM ammonium bicarbonate, pH 8.0

(ABC). Each wash consisted of bead resuspension in 500 μL of each buffer, pelleting of beads at 500 × g for 30 s and aspiration of supernatant. On-bead digestion was performed by resuspending beads in 100 μL of ABC containing 1 μg of sequencing grade trypsin (T6567, Sigma-Aldrich). Samples were gently mixed at 37°C overnight. Samples were spiked with 1 μg of fresh trypsin and digested further for 3 h. The supernatant, containing digested peptides, was transferred to new tubes. Beads were washed twice with HPLC-grade water to wash off peptides, and these were pooled with the collected supernatant. Peptides were vacuum centrifuged until dry.

Mass spectrometry acquisition

Each sample was resuspended in 5% formic acid and loaded onto an equilibrated high-performance liquid chromatography column (800 nL/min). Peptides were eluted with a 90 min gradient generated by a Eksigent ekspert nanoLC 425 (Eksigent, Dublin CA) nano-pump and analyzed on a TripleTOF 6600 instrument (AB SCIEX, Ontario, Canada).

The MS acquisition method has been described previously on identical instrumentation.[71] The gradient was delivered at 400 nL/min and consisted of three steps: sample delivery, column cleanup and column equilibration. The gradient used to pass sample over the column took place over 90 min starting with 2% acetonitrile (ACN) + 0.1% formic acid (FA) and ending with 35% ACN +0.1% FA. Cleanup was performed by passing 80% ACN +0.1% FA over the column for 15 min, and the column was equilibrated back to 2% ACN +0.1% FA over 15 min.

Instrument calibration was performed on bovine serum albumin reference ions to adjust for mass drift and verify peak intensity before samples were analyzed in data-dependent acquisition (DDA) mode. One 250 ms MS1 TOF (time of flight) survey scan (over mass range 400–1800 Da) was performed and was followed by 10 × 100 ms MS2 candidate ion scans (100–1800 Da). Ions that exceeded a threshold of 300 counts per second and had a charge of 2+ to 5+ were selected for MS2. Precursors were excluded for 7 s after one occurrence.

Data-dependent acquisition data search

The ProHits laboratory information management system was used to analyze proteomics data.[72] WIFF files were converted with the WIFF2MGF converter and to a mzML format using ProteoWizard (V3.0.10702) and the AB SCIEX MS data converter (V1.3 beta). Converted files were searched with Mascot (2.3.02)[79] & Comet (2016.01 rev.2).[80] Spectra were searched against a collection of 72,482 entries comprised the following: human and adenovirus sequences (version 57, January 30th, 2013), common contaminants [Max Planck Institute (http://maxquant.org/contaminants.zip) & Global Proteome Machine (GPM; ftp://ftp.thegpm.org/fasta/cRAP/crap.fasta)], reversed sequences, bait tags (e.g., BirA or GFP) and streptavidin. Search parameters were set to search for trypsinized peptides allowing for two missed cleavages. For precursors, a mass tolerance of 35 parts per million was set, and peptides of +2 to +4 charges were allowed with a tolerance of ±0.15 amu for fragment ions. Variable modifications included deamidated asparagine and glutamine as well as oxidized methionine. Search results were analyzed with the Trans-Proteomic Pipeline (v4.7 POLAR VORTEX rev 1) and iProphet pipeline.[81]

SAINT analysis

An iProphet probability score >0.95 and more than two unique peptides were required for protein identification. SAINTexpress [version 3.6.1[82]] was used to score proximity interactions from DDA data using default parameters. Bait runs, run in biological duplicate, were compared against four negative control runs consisting of two miniTurbo-eGFP-only samples and two untransduced MCF10A samples. Control runs were not compressed for this analysis. Preys with a Bayesian false discovery rate <5% were considered high-confidence proximity interactions. gProfiler (https://biit.cs.ut.ee/gprofiler/gost) was used to calculate enrichment of GO cellular component terms.

RNA *in situ* hybridization and immunofluorescence of GCRC1735

FFPE tissue was deparaffinized and underwent heat-mediated antigen retrieval in citrate buffer pH6.0 or EDTA buffer pH9.0. Slides were blocked with Power Block for 5 min at room temperature, and incubated with the primary antibody for 30 min at room temperature followed by washing with TBST (3 × 3min). Slides were incubated with secondary antibody-HRP for 30 min at room temperature, washing with TBST 3 × 3min and stained with Opal fluorophore working solution for 10 min. This was followed by heat-mediated antibodies stripping to remove the primary and secondary antibodies in order to repeat additional rounds for labeling with other primary antibodies. The primary antibodies are against Ki67 (Ventana #790–4286) and Pan-Keratin (Cat# 760–2595, Ventana). The antibody specificity and dilution were tested before multiplex assay. Nuclei were stained with 0.5 ng/mL DAPI for 5 min at room temperature and counterstaining was done with Harris' hematoxylin. RNA *in situ* hybridization was performed using the RNAscope 2.5 HD Assay (cat#322360. ACD Bio) according to the manufacturer's instruction on FFPE PDX section. The probes used are Hs-RBPJ (cat#448661), the positive control Hs-PPIB housekeeping gene and the negative control dapB. Slides were imaged with an LSM800 confocal microscope (Zeiss). Brightfield slides were scanned using Aperio-XT slide scanner (Aperio). Visual inspection was used to classify cells into RBPJ or Ki67 high and low classes. Due to low number of RBPJ high expression cells a field with equal number of cells of both RBPJ expression classes was used for the quantification.

Colony formation assay of *C4orf19* loss-of-function CRISPR-Cas9 mutant cells

sgRNA sequences were obtained from TKOv3[74]: (5′-3′) sg-LacZ GCCCGAATCTCTATCGTGCGG, sg1-C4orf19-TGTGGACGAG GATGCAGCGG, sg2- C4orf19-AACAGGCAGGAGGATACCCA and sg3-C4orf19-GCAGCAGGACAGCTGATCCA. sgRNA were ordered from IDT and combined with purified Alt-R Cas9 (IDT) to generate CRISPR-Cas9 RNPs. RNA oligos were resuspended in IDT Duplex buffer to 200 μM and 5 μL of the suspension was mixed with 5 μL of 200 μM tracrRNA, incubated at 95°C for 5 min

and cooled to room temperature. For *LacZ*, RNP complex was prepared by mixing 2.1 μL PBS, 1.2 μL crRNA:tracrRNA duplex and 1.7 μL Alt-R Cas9 enzyme (61 μM stock) and incubated at room temperature for 10–20 min. For *C4orf19*, separate RNP complexes were prepared by mixing 0.7 μL PBS, 0.4 μL crRNA:tracrRNA duplex and 0.6 μL Alt-R Cas9 enzyme (61 μM stock) per sgRNA and incubated at room temperature for 10–20 min. RNPs were combined, 200,000 MCF10A cells were trypsinized per protocol and nucleofected using Lonza Nucleofector as per manufacturer's instructions. Briefly, MCF10A cells were resuspended in 100 μL of Nucleofector solution SE and 20 μL cell suspension were combined with 5 μL RNP and 1 μL of 100 μM Alt-R Cas9 electroporation enhancer. Then, 25 μL of cell suspension was transferred to a nucleovette cuvette and nucleofected using 4D X-unit Nucleofector, program DS-138. 75 μL of warm media were added to the nucleofected cell suspension and transferred to a 6-well plate. Media was changed the next day and cells were allowed to growth for 4 days to allow Cas9 editing to go on for completion. The *C4ORF19* edited locus comprising three target sites was PCR amplified using the following primers C4orf19_PCR_F (5′-3′) ACTGTCTCGCTCTGTCATC and C4orf19_PCR_R (5′-3′) GGGACTTCTGCTCTACTGTG and sequenced using Sanger sequencing and the following primer C4orf19_seq (5′-3′) GCGTGAGCCACCAAACAT to verify successful editing. DECODR[83] was used to deconvolve the sequencing data resulting in the gene editing efficiency of 87.3%.

For the colony formation assay 1,000 cells of each Cas9-edited population were seeded in 6-well dish in two technical replicates and three biological replicates. Cells were allowed to grow for 9 days to form colonies, followed by fixation with 1 mL of 4% PFA for 20 min with agitation, four washes with PBS and staining with 1% crystal violet (20% methanol) for 10 min. Once images were acquired using a camera, crystal violet stain was dissolved using 2 mL of 10% acetic acid, incubated at room temperature for 20 min with agitation and absorbance was measured at 590 nm as a proxy or cell growth and normalized relative to control.

C4orf19 immunofluorescence and quantification

C4orf19 immunofluorescence staining: cells were seeded in 24-well plate with coverslips until they reached 80–90% confluence. Then, they were fixed in 4% paraformaldehyde (20 min), permeabilized with 0.2% Triton X-100 (10 min), blocked with 2% BSA (30 min), and then incubated with Anti-C4orf19 primary antibody (1:100, GeneTex, GTX106538) (1 h). The primary antibody was visualized with a fluorescent secondary antibody conjugated to Alexa Fluor 488 raised in goat (1:1000, Invitrogen A21206) (1 h). Nuclei were counterstained with 0.25 ng/mL DAPI (5 min). All steps were performed at room temperature. Images were acquired on the Nikon C2/TIRF confocal laser scanning microscope (Nikon), using a 63X objective.

Fiji (v.2.3, NIH) was used to analyze subcellular localization of C4ORF19 using a custom macro. The DIC image was used to manually outline each cell; DAPI was used to create a nuclear mask and GFP channel was used to quantify C4ORF19 subcellular localization. The macro makes bands of ∼3 μm from the edge of a cell outline into the middle of the cell, and measures the mean intensity, total amount of signal, the proportion of the cell's total area that is in this band, the proportion of the cell's total signal that is in this band, and the ratio of the signal-to-area. The ratio of the signal-to-area is above 1, if there is a greater proportion of the signal in that band than might be expected based solely upon area. The outer band representing ∼25% of the cell area was compared to the remainder of the cell to quantify the protein abundance of C4orf19 in the cell periphery compared to the cytoplasm.

Coexpression of the C4orf19-PDCD10-GCKIII module

cBioPortal was used to access the Cancer Cell Line Encyclopedia (Broad, 2019) mRNA expression (RNA Seq RPKM) (1156 samples) dataset. mRNA expression z-scores relative to all samples (log RNA Seq RPKM) were used at default settings. Pairwise correlation between genes was calculated as a measure of co-expression using Spearman correlation coefficient, $p < 0.05$.

Single cell RNA sequencing

Breast mammoplasty reduction epithelial single-cell suspensions were washed three times in PBS with 0.04% BSA. An aliquot of cells was used for LIVE/DEAD viability testing (Thermo Fisher Scientific). Single-cell libraries were generated using the Chromium Controller and Single Cell 3′ Library & Gel Bead Kit v3 and Chip Kit (10x Genomics) according to the manufacturer's protocol. Briefly, cells suspended in reverse transcription reagents, along with gel beads, were segregated into aqueous nanoliter-scale gel bead-in-emulsions (GEMs). The GEMs were then reverse transcribed in a T1000 Thermal cycler (Bio-Rad) programmed at 53°C for 45 min, 85°C for 5 min, and hold at 4°C. After reverse transcription, single-cell droplets were broken and the single-strand cDNA was isolated and cleaned with Cleanup Mix containing DynaBeads (Thermo Fisher Scientific). cDNA was then amplified with a T1000 Thermal cycler programmed at 98°C for 3 min, 12 cycles of (98°C for 15 s, 63°C for 20 s, 72°C for 1 min), 72°C for 1 min, and hold at 4°C. Subsequently, the amplified cDNA was fragmented, end-repaired, A-tailed and index adaptor ligated, with SPRIselect Reagent Kit (Beckman Coulter) with cleanup in between steps. Post-ligation product was amplified with a T1000 Thermal cycler programmed at 98°C for 45 s, 12 cycles of (98°C for 20 s, 54°C for 30 s, 72°C for 20 s), 72°C for 1 min, and hold at 4°C. The sequencing-ready library was cleaned up with SPRIselect and quantified by qPCR (KAPA Biosystems Library Quantification Kit for Illumina platforms). 200 p.m. of sequencing libraries were loaded on an Illumina HiSeq instruments (see single-cell RNA sequencing analysis section) and ran using the following parameter: 26 bp Read1, 8 bp I7 Index, 0 bp I5 Index and 98 bp Read2.

Single-cell RNA sequencing analysis

Two samples were sequenced using the Chromium single cell 3′ RNA-seq on 0.5 lane the NovaSeq S1 instrument, for a total of 492,626,914 reads, and 327,762 reads per single-cell (saturation 89.2%). Alignment against the human GRCh38 genome was

performed using Cell Ranger Pipeline version 3.0.1. The Ensembl annotation for GRCh38 (release 93) was used, keeping only the genes with the biotypes protein_coding, lincRNA and antisense. Empty GEMs containing only background reads were discarded by the pipeline and bar code errors resulting from sequencing were corrected if they contained only one mismatch by assigning them to the closest available bar code, or discarded otherwise, resulting in 1,503 GEMs containing cells. Alignment quality was controlled by assessing the proportion of reads mapping confidently to the transcriptome (52.4%). The total number of genes detected (>1 mapped read) was 20,541, with a median of 880 per cell. The R package Seurat (v3.2.3) was used to analyze the single-cell RNA-seq data (Satija et al., 2015). Cells with over 12% mitochondrial content, over 40,000 UMIs, or less than 500 UMIs were discarded. Gene counts were normalized

to a total of 10,000 UMIs for each cell, and transformed to a natural log scale. Counts were then adjusted for library size and mitochondrial proportions. Heat Digestion Stress Response Gene Set as previously reported[84] was identified and removed. Cell types were annotated using previously defined markers[84] (Figure S4A). Cells in the breast epithelial cell cluster were used as a baseline of normal gene expression for inferring copy number described below.

Inferring copy number aberrations from scRNA-seq

Copy number profile was inferred from scRNA-seq data as previously described.[33] Briefly, the gene expression was normalized to ensure cells are comparable, whereby the Trimmed-Mean M normalization rescale expression in a cell by a factor to match that of a control cell. Consecutive genes were merged to form "bins" with a minimum average expression. Each bin was normalized across cells to produce a *Z* score which was computed by subtracting the average expression across all the cells and dividing by the standard deviation. The normalized expression was smoothed using a rolling median approach: the expression in each bin was replaced by the median expression of the surrounding bins in the cell. Specifically, the rolling median was run-in windows of size 5, i.e., the bin of interest and two bins on each side. The smoothed *Z* score was winsorized to be within [-3,3] to further reduce the effect of single-gene outliers. After this step, the score is centered on 0 and positive (negative) values support a higher (lower) copy number than in the majority of the cells. A principal component analysis (PCA) was run on the smoothed Z-scores. To minimize the effect of cell cycle, the PCA was run on non-cycling cells and all the cells projected on this principal components (PCs).

Community detection was performed by the Louvain algorithm on a cell network built from the top PCs. A tSNE was run on the top 20 PCs. To build the cell network, we first identify the K-nearest neighbors of each cell based on the Euclidean distance D in the PC space. The K-nearest neighbor cells are linked in the network with a weight defined as 1/(1 + D). The Louvain community detection was then run on this cell network. Gamma resolution parameter with a high mean Rand Index across the runs and/or a low Rand Index variance was used. Copy-number aberrations are called at the community level to increase the sensitivity to shorter aberrations. Meta-cells were constructed by combining the expression of randomly selected cells in a community. We created multiple meta-cells for each community and looked for consistent CNA signal in all meta-cells. The expression in each meta-cell was normalized similarly as for the CNA-based community detection: normalization per cell, merging into expressed bins, *Z* score computation, and smoothing. The *Z* score was computed relative to a specific baseline, e.g., cells identified as normal that were isolated from mammoplasty reduction. CNA were called using an HMM with three states: neutral copy number, loss, and gain. A Gaussian mixture HMM was capable of segmenting together the multiple meta-cells from a community. Short copy-number segments are filtered, for example if spanning less than 5 consecutive bins, as they could result from single genes with strong expression differences. A Wilcoxon test was performed to assess the significance of each loss/gain segment by comparing the expression within the segment with the expression in nearby "neutral" segments.

Frequency of cells inferred to harbor chromosome 4p deletion or copy neutral state across different cellular clusters was compared for cells with distinct transcriptional programs. The "transcriptional program" category received a count for any combination in which a cell belonged both to a specific inferred copy number community and a specific gene expression cluster. The fold increase over the background fraction of all cells in a specific gene expression cluster was calculated. Significance was assessed with a hypergeometric test.

WGS analysis

Bulk WGS data for PT and PDX including BAM generation, Manta calls for structural variants and Mutect2 calls for somatic mutation variants for were obtained from a previous study.[29]

Timing analysis using bulk WGS data for basal breast cancer PT/PDX panel

CNA profiles

CNA profiles were obtained using Battenberg (v2.2.9),[85] integrating SV calls from Manta and correcting logR for both GC content and replication timing. SNP phasing was performed using Beagle 5.1 (18May20.d20). To better capture LOH-related events in PDX samples, because BAF in pure samples tends to be highly squished and might be misinterpreted, purity was artificially decreased by pulling allele counts from both germline and PDX and was set back to 100% when checking sample purity with SNV information from Mutect2. All CNA profiles were manually examined and quality checked (e.g., homozygous deletions, superclonal peaks, purity estimates, etc.). Whole-genome doubling (WGD) information was assessed using the relationship between the fraction of the genome with LOH and ploidy, as in PCAWG studies.[86]

CNA clustering

Clustering of CNA profiles was performed using MEDICC2 (v0.3).[87] Genomic regions of more than 500kb and covered in all samples for a given patient were considered, with *cn_a* and *cn_b* defined as the major and the minor allele, respectively. The copy-number state of the most abundant subclone was selected for subclonal CNAs. Since WGDs were clonal events, we defined reference normals with a 1 + 1 baseline in samples without WGD and 2 + 2 with WGD so the output was WGD-aware.

Subclone trees

Subclonal compositions were assessed using DPClust (v2.2.8)[85] and SNVs information from Mutect2 calls, leveraging principles of reconstructing subclone trees.[88] Small and noisy clusters (<5% of total SNVs) breaking the pigeonhole principle were discarded from the final trees.

Genomic event timing

Clonal copy number gains were timed using an approach similar to that outlined in a previous study.[17] A posterior distribution over SNV multiplicities was measured using the emcee sampler (https://arxiv.org/abs/1202.3665) with a prior that corresponded to a uniform distribution over gain timing. For each segment we ran 30 independent chains for 2000 steps with 1000 burnin steps. The posterior distribution over SNV multiplicities was converted into a distribution over gain timing.

The timing of the WGD for WGD tumors was measured by jointly timing all the gains that resulted in a major copy number state of two. For WGD samples, the timing of gains leading to major copy number three and four states were measured with equal prior probability on a gain occurring before or after the WGD. The relative likelihood of pre- or post-WGD gains was calculated by measuring the similarity between the segment WGD timing distribution measured with the route compared to the sample-wide WGD timing distribution. For gain regions with major copy number four, the timing of the average of the two post-WGD gains was measured as the system is underdetermined. Only gained regions with a major copy number of up to four in WGD tumors and two in non-WGD tumors were timed. The gained regions also needed to have at least 10 SNVs and a minor copy number of no more than two. The timing of SNVs in key genes was measured using MutationTimeR.[17]

The PDXs were used to refine our timing of events in the primary tumor. If an event was identified as clonal in the primary tumor, but was not found in the PDX, we reclassified the event as subclonal in the primary as the event was likely not present in the cells from the primary that seeded the PDX.

League model

A league representing a timeline of genomic events aggregated across tumors were produced from our timing data using a league modeling approach similar to that outlined in previous studies.[17,75] Briefly, the aggregate timing of the events is determined by running a scoring process where the earliest events accumulate the higher score. This is achieved by initialising each genomic event with a score of zero. We then sample the relative timing for each possible pair of genomic events from the subset of individual tumor timelines in our cohort that contain both events. The earlier event has its score increased by one and the later event decreased by one. If the relative timing of the event pair cannot be distinguished, or if no sample has both events, the score for both events is kept the same. After each possible pair of events is considered, the events are ranked according to their score. This process is repeated 100 times to achieve a distribution over the ranks.

Real-time timing

A real-time estimate of WGD and the emergence of the MRCA was achieved using the approach outlined in a previous study.[17] Instead of evaluating the timing using both a branching and linear subclonal structure[17] we used the structure inferred from our subclone tree reconstruction.

scDNA-seq

Basal breast cancer PDX-derived GCRC1735 single-cell suspensions were washed three times in PBS with 0.04% BSA. An aliquot of cells was used for LIVE/DEAD viability testing (Thermo Fisher Scientific). Single-cell DNA libraries were generated using the Chromium Single Cell DNA Reagent Kit (10X Genomics) according to the manufacturer's protocol. Briefly, an appropriate volume of cell suspension for targeting 500 cells were added to the Single Cell Bead Mix then loaded onto a Chromium Chip C, along with CB polymer. The resultant Cell-Bead was allowed to polymerize overnight shaken at 1000rpm. The encapsulated cells were then lysed and its genomic DNA was NaOH denatured. The Cell-Bead along with a reaction mix and Gel-Bead were loaded onto the Chromium D Chip to generate gel bead-in-emulsions (GEMs) on the Chromium Controller. An ideal GEM will contain reaction mix, one Cell-Bead and one Gel-Bead. The GEMs were incubated in a T1000 Thermal cycler (Bio-Rad) programmed at 30°C for 3hour, 16°C for 5 h, 65°C for 10 min, and hold at 4°C. Then the GEMs were broken and its amplified DNA were isolated using Dynabeads MyOne Silane beads followed by a SPRIselect cleanup. DNA was quantified on a Caliper Labchip (Beckman Coulter) using High Sensitivity DNA Assays. The DNA was converted to sequence ready library by fragmentation, end-repaired, A-tailing, index adaptor ligated and index PCR with SPRIselect clean ups in between. Four samples were sequenced on 2 lanes the Illumina HiSeqX instrument, for a total of 3,726,525,082 reads, and 505,266 reads per single-cell (saturation 15%).

scDNA-seq data processing

Sequencing data was processed by using 10X Cell Ranger DNA pipeline to generate a raw bam for each sample. Briefly, the reads were aligned to the human reference genome build 38 (GRCh38) by using BWA and then converted to sorted BAM. The bam file was demultiplexed into individual bam files by using in-house python script to represent the sequencing reads from each single cell.

Poorly mapped reads with mapping quality <25 were filtered out by using SAMtools. PCR duplicates were removed by using Picard. Noisy cells detected by 10X Cell Ranger DNA pipeline with depth independent MAPD statistically higher than the sample distribution (with p value <0.01) and low ploidy confidence were excluded. We also filtered out cell outliers with large Lorenz curve area.[89]

scDNA-seq SNV analysis

To create a pseudo-bulk sample, single-cell DNA (scDNA) samples were merged while preserving the cell of origin information. This was achieved by incorporating the cell of origin information into the read group field of each read. The pseudo-bulk sample was then processed as a standard whole-genome sequencing (WGS) sample using the tumor_pair pipeline from Genpipes.[90] Somatic variants were generated using Mutect2 and were utilized for single-nucleotide variant (SNV) fishing in individual cells. To reduce the false positive rate during the fishing process, we excluded indels and retained only high-quality somatic variants (TLOD $\geq$ 40). For each cell (represented by each read-group in the pseudo-bulk), we extracted the base distribution at each selected somatic position using BVAtools basefreq (https://bitbucket.org/mugqic/bvatools/src/master/). Cell-specific somatic variants were determined by comparing the extracted base frequency with the expected variant allele detected by Mutect2. In order to ensure robustness, cell-specific somatic variants were excluded if they were not genotyped in at least 10 different cells.

Building the clone tree and assigning SNVs to clones using heuristics

SNVs were used to build the clone tree for GCRC1735 primary and PDX tumors. SNVs were independently called in four datasets: primary tumor bulk (PT_bulk), PDX bulk (PDX_bulk), single-cell samples 1 and 2 pseudobulk (SCS12) and single-cell samples 3 and 4 pseudobulk (SCS34). After examining the patterns of SNVs presence and absence in the samples, and based on the known ancestral relationships among the sample, the following heuristics were developed to assign mutations to clones (Figure S3B). First, if a mutation was present in all datasets, it was deemed a clonal mutation. Second, if a mutation was present in PT_bulk and SCS12 and not present in SCS34 or PDX_bulk, then it was assigned to subclone 1. Third, if a mutation is present in PT_bulk, PDX_bulk and SCS34 but not in SCS12, then it is set to subclone 2. Fourth, if a mutation is present only in PDX_bulk and SCS34 then it is set to subclone 3. Due to the low count of unique mutations to PT_bulk or PDX_bulk, we did not attempt to further define smaller subclones present in these samples.

Inference of haplotype-specific copy number profiles in single cells using CHISEL

CHISEL[91] is a tool that infers haplotype-specific copy number profiles of each cell in a low coverage single-cell sequencing dataset. This was used on the single-cell datasets to determine copy number profiles for subclones 1 and 3. To run CHISEL we first installed the package and dependencies as instructed on their GitHub page. Germline SNPs were called using bcftools' mpileup and call methods on the normal sample.baf file. These SNPs were phased using the Michigan Imputation Server (MIS). As required for MIS input, the SNP calls were separated by chromosome into separate.bcf files, sorted by genomic position, and uploaded to their server. For phasing on MIS, we used as arguments: reference panel HRC r1.1 2016 (GRCh37/hg19), array build GRCh38/hg38, phasing Eagle v2.4, and mode Quality control and phasing only. The MIS results were downloaded as a set of chromosome-specific.vcf files and merged. The X chromosome was omitted during this merge as, at the time of running, sex chromosomes were not permitted in CHISEL's input. The MIS outputs phased SNP data using hg19 reference genome coordinates. Therefore, these coordinates were lifted over to the hg38 reference genome. This was done using picardtool's LiftoverVcf function with the requisite UCSF chain file. CHISEL also requires a.bam file for the single-cell sequencing data. In CHISEL, haplotypes are categorized as maternal or paternal arbitrarily, and so single-cell datasets could not be input to CHISEL separately. Instead, all single-cell data files were merged into a single.bam file and used as input. With these phased germline SNPs and the single-cell.bam file, CHISEL was run with default settings. This process was repeated using a different seed to confirm reproducibility.

Inferring the most recent common ancestor (MRCA) copy number profile

The results from CHISEL reveal two predominant subclones, each defined by the PDX line the cells are derived from. To estimate the haplotype-specific copy number profile of their MRCA, we developed and applied the following heuristics. First, if an allele's copy number was the same between the two subclones, that allele's copy number was set to the same for the MRCA. Second, if there was a LOH event in one subclone but not the other, the MRCA copy number state was set to contain the lost allele. Third, in other regions of differing copy number, if there was an adjacent region with shared copy number between the two subclones, and that region's copy number matched that of one of the two mismatched copy numbers, then the MRCA copy number for the differing region was set to be the same as the matching neighbor. If none of the previous three heuristics apply, then the MRCA copy number is set to be the minimum of the two subclonal copy numbers.

QUANTIFICATION AND STATISTICAL ANALYSIS

Statistical parameters, including number of biological replicates, sample sizes, algorithm parameters are reported in the method details and the figure legends.

Cell Reports, Volume *43*

Supplemental information

Evolution of chromosome-arm aberrations

in breast cancer through genetic network rewiring

Elena Kuzmin, Toby M. Baker, Tom Lesluyes, Jean Monlong, Kento T. Abe, Paula P. Coelho, Michael Schwartz, Joseph Del Corpo, Dongmei Zou, Genevieve Morin, Alain Pacis, Yang Yang, Constanza Martinez, Jarrett Barber, Hellen Kuasne, Rui Li, Mathieu Bourgey, Anne-Marie Fortier, Peter G. Davison, Atilla Omeroglu, Marie-Christine Guiot, Quaid Morris, Claudia L. Kleinman, Sidong Huang, Anne-Claude Gingras, Jiannis Ragoussis, Guillaume Bourque, Peter Van Loo, and Morag Park

Figure S1

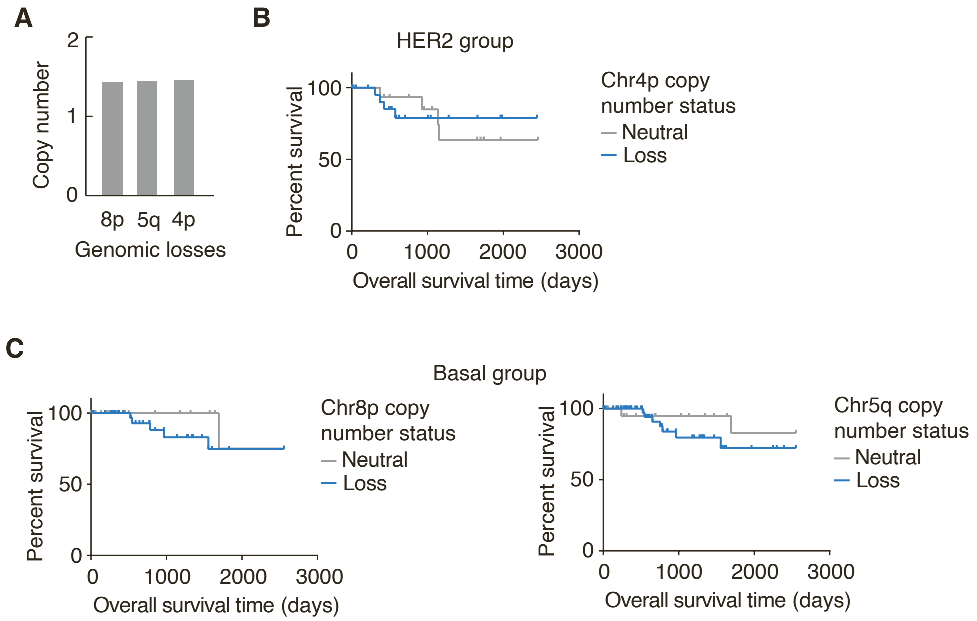

Figure S1. Recurrent large chromosomal deletions in breast cancer. (A) Copy number was obtained from the TCGA segmented mean showing that frequently recurrent large chromosomal deletions in basal breast cancer are hemizygous, n = 91. **(B)** Overall survival of Her2 breast cancer patients with copy neutral and deletion status of chr4p shows no difference between groups, n = 55. **(C)** Overall survival of basal breast cancer patients with copy neutral and deletion status of chr8p and chr5q shows a trends towards a worse survival of patients with these chromosome arm losses (chr 8p loss $p = 0.5$, chr5q loss $p = 0.4$ as assessed by long rank test; n = 91).

Figure S2

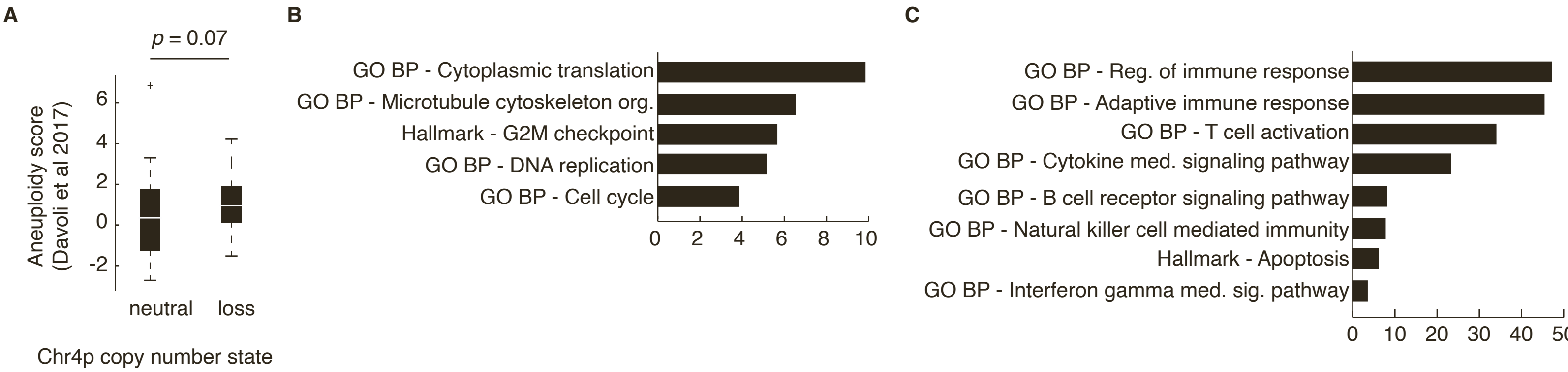

Figure S2. Aneuploidy in basal breast cancer with different chr4p copy number states. (A) Aneuploidy score as quantified by Chrom.Arm.SCNA.Level median reported by (Davoli et al)20 shows no statistically significant difference in aneuploidy between chr4p copy neutral vs deletion basal breast cancer samples. Significance was assessed using Wilcoxon rank sum test Gene Set Enrichment Analysis (GSEA) showing representative terms that are enriched for genes displaying **(B)** elevated or **(C)** decreased expression due to chr4p loss in TCGA basal breast cancer after correcting for aneuploidy as scored in **(A)**.

Figure S3

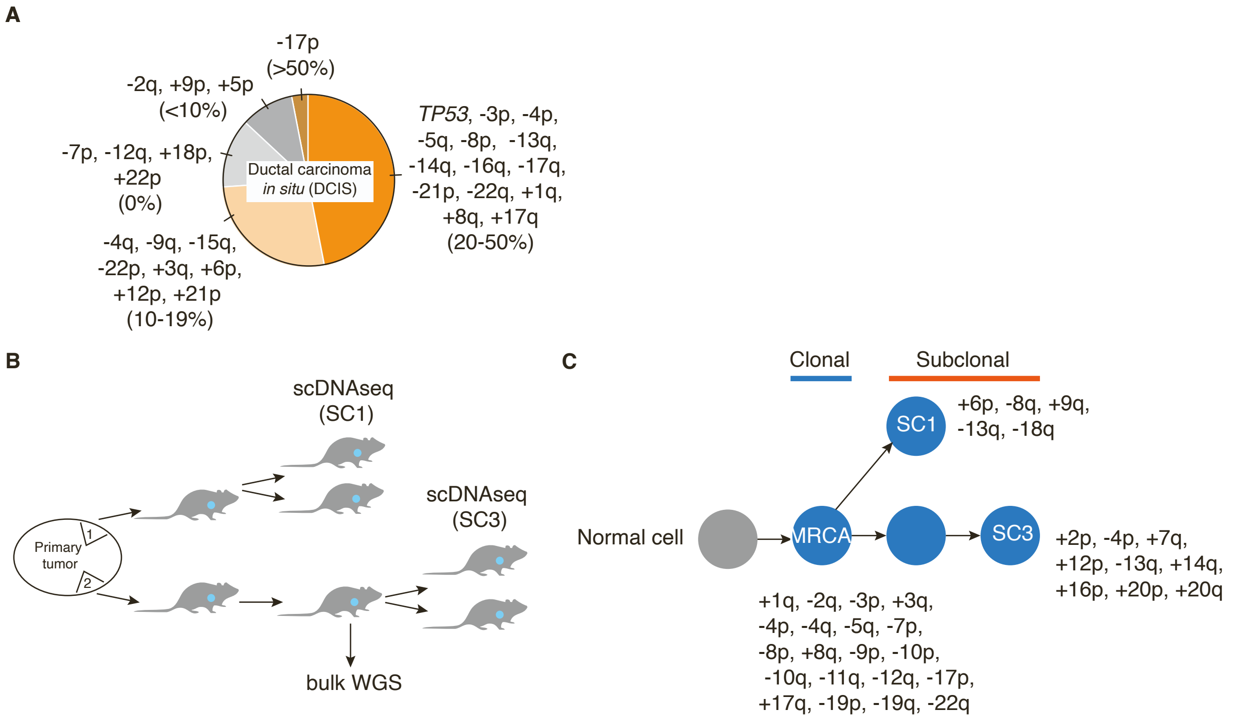

Figure S3. Frequency of genomic events in other datasets. (A) Genetic events from clonal and variable/constant regions of the timing analysis presented in Figure 2B were detected in Ductal Carcinoma *in situ* (DCIS) from a previous study (Lips et al)31. Frequency of patient samples from a total of 95 is shown in brackets. **(B)** Schematic of PDX generation, which was used for bulk WGS and scDNAseq. Single cell DNA sequencing was conducted on four GCRC1735 PDX samples. Two different locations within the primary tumor were biopsied, cryopreserved and propageted in NOD-SCID mice. Two mice were engrafted using a fragment derived from one location from passage 2 in the PDX and two mice were engrafted using a fragment drived from another location from passage 3 in the PDX. **(C)** CHISEL (Zaccaria et al)91 was used to generate an evolutionary timeline showing that chr4p loss is an early event in basal breast cancer PDX progression.

Figure S4

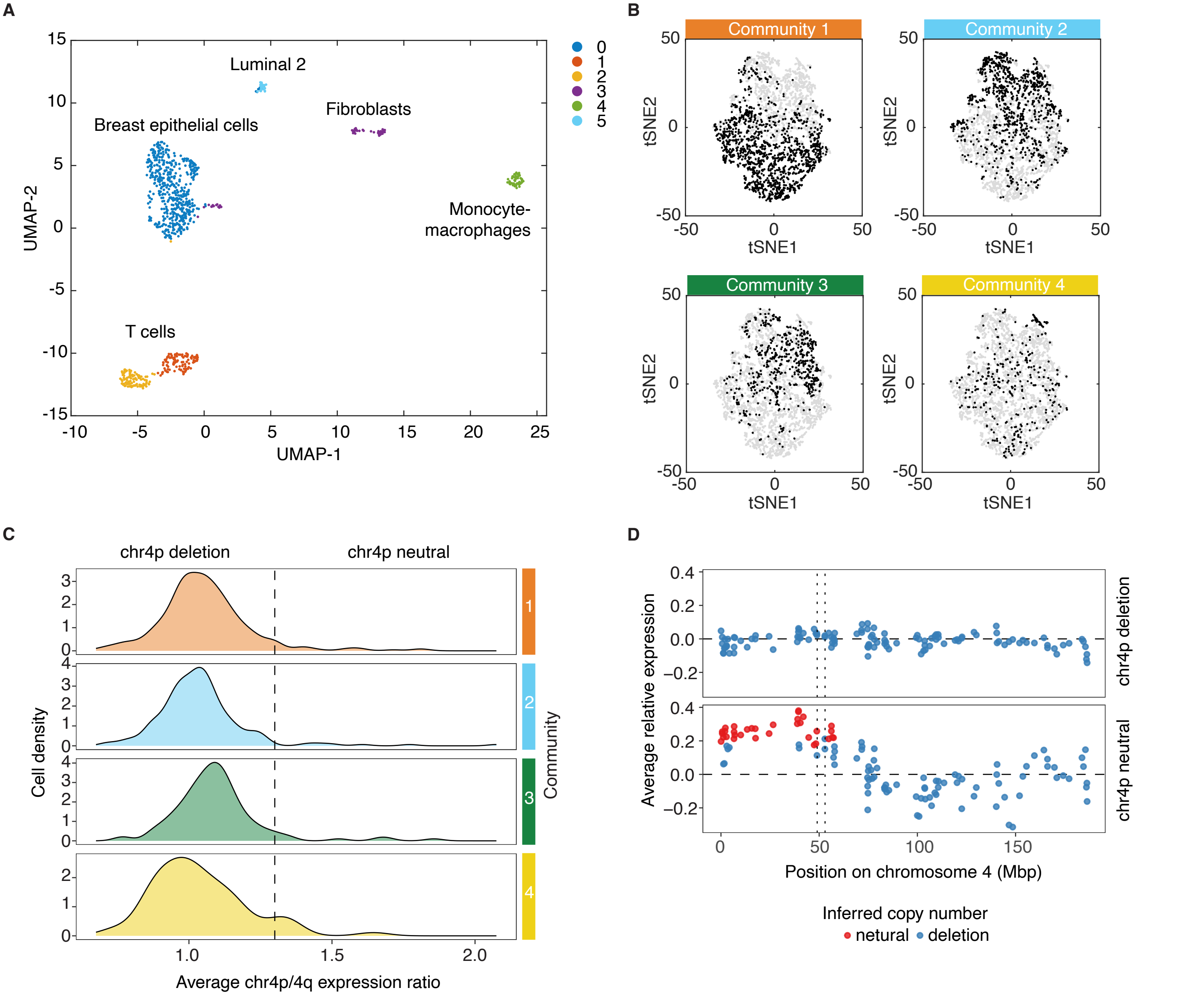

Figure S4. Inferring copy number aberrations from scRNAseq. (A) scRNAseq of normal breast epithelial cells. UMAP plot of single cell RNA seq data from two reduction mammoplasty samples. Clusters were annotated using previously defined cell type markers. **(B)** Overlapping inferred copy number communities with scRNAseq gene expression clusters. scRNAseq map as reported in a previous study is used to annotate single cells with inferred copy number communities as analyzed in this study. Single cells coloured in black belong to the specifiied community. **(C)** To infer chr4p-copy neutral state, we computed the average expression between regions in chr4p and 4q (x-axis). A ratio higher than 1 suggests more copies of chr4p, indicating chr4p copy neutral cells since cells in this tumor harbor chr4q loss. The cells are grouped by the CNA communities depicted in Fig. 4A. The dotted line shows the thresholds used to separate chr4p neutral cells from chr4p loss cells. **(D)** Inferred copy number aberrations shows a clear and consistent signal for chr4p in cells as computed in panel C. The x-axis shows the position across chromosome 4, with the centromere marked with vertical dotted lines. The y-axis shows the average expression of the region relative to the expression along chr4p across all cells attributed to genomic copy number state (higher: neutral, lower: deletion).

Figure S5

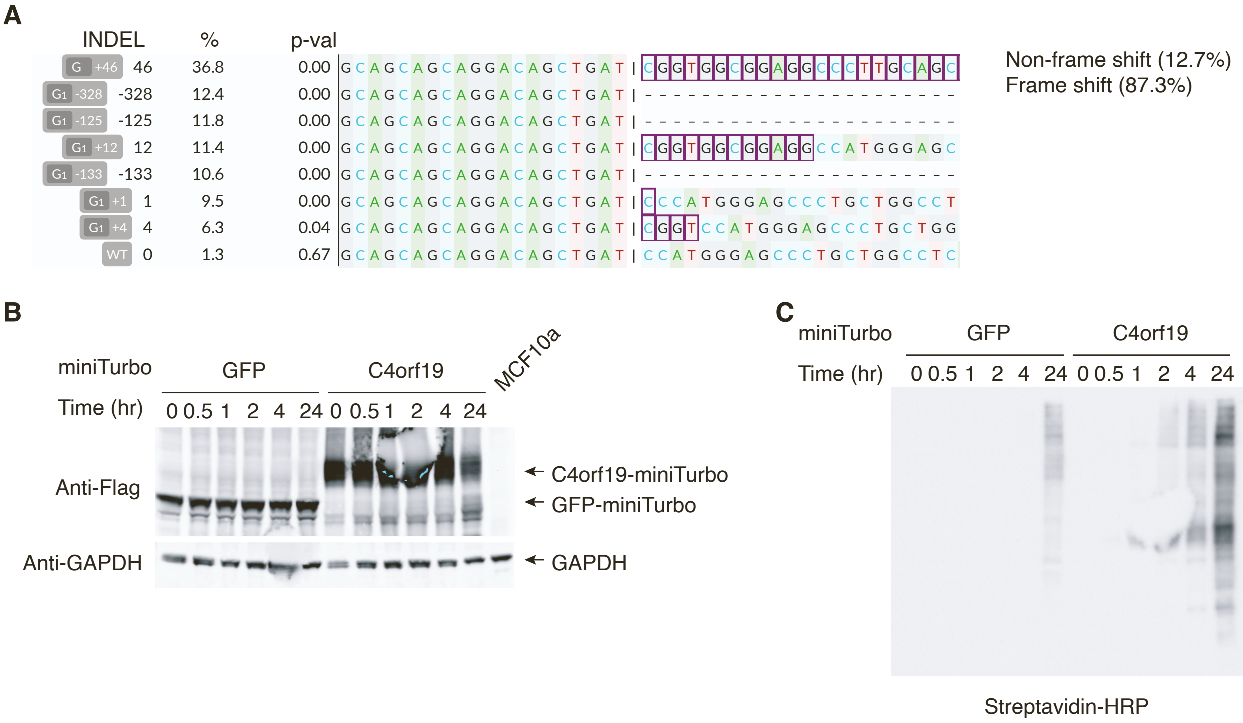

Figure S5. *C4orf19* functional characterization. (A) *C4orf19* CRISPR-Cas9 gene editing efficiency as reported by DECODR. INDEL refers to indel distribution with percentage of reads with a specific indel noted in (%) with the corresponding p-value. **(B)** miniTurboID C4orf19 protein bait expression in MCF10a. MCF10a cells stably expressing miniTurboID bait proteins: C4orf19-3XFLAG-miniTurbo and GFP-3XFLAG-miniTurbo control were induced with 0.5 μg/ml doxycycline for designated time. Protein bait expression was assessed using Anti-Flag antibody. GAPDH protein level served as the loading control, n = 3. **(C)** Biotinylation level. Biotin labeling was induced with 0.5 μg/ml doxycycline and 40 μM biotin for designated time. Optimal biotinylation was achieved 4 hr post induction, n = 3.
